# MHJ_0461 is a multifunctional leucine aminopeptidase on the surface of *Mycoplasma hyopneumoniae*

**DOI:** 10.1098/rsob.140175

**Published:** 2015-01-14

**Authors:** Veronica M. Jarocki, Jerran Santos, Jessica L. Tacchi, Benjamin B. A. Raymond, Ania T. Deutscher, Cheryl Jenkins, Matthew P. Padula, Steven P. Djordjevic

**Affiliations:** 1 The ithree institute, University of Technology, Sydney, PO Box 123, Broadway, New South Wales 2007, Australia; 2 Proteomics Core Facility, University of Technology, Sydney, PO Box 123, Broadway, New South Wales 2007, Australia; 3 NSW Department of Primary Industries, Private Bag 4008, Narellan, New South Wales 2567, Australia

**Keywords:** adhesin, DNA-binding protein, plasminogen-binding protein, heparin-binding protein, leucine aminopeptidase, moonlighting protein

## Abstract

Aminopeptidases are part of the arsenal of virulence factors produced by bacterial pathogens that inactivate host immune peptides. *Mycoplasma hyopneumoniae* is a genome-reduced pathogen of swine that lacks the genetic repertoire to synthesize amino acids and relies on the host for availability of amino acids for growth. *M. hyopneumoniae* recruits plasmin(ogen) onto its cell surface via the P97 and P102 adhesins and the glutamyl aminopeptidase MHJ_0125. Plasmin plays an important role in regulating the inflammatory response in the lungs of pigs infected with *M. hyopneumoniae*. We show that recombinant MHJ_0461 (rMHJ_0461) functions as a leucine aminopeptidase (LAP) with broad substrate specificity for leucine, alanine, phenylalanine, methionine and arginine and that MHJ_0461 resides on the surface of *M. hyopneumoniae*. rMHJ_0461 also binds heparin, plasminogen and foreign DNA. Plasminogen bound to rMHJ_0461 was readily converted to plasmin in the presence of tPA. Computational modelling identified putative DNA and heparin-binding motifs on solvent-exposed sites around a large pore on the LAP hexamer. We conclude that MHJ_0461 is a LAP that moonlights as a multifunctional adhesin on the cell surface of *M. hyopneumoniae*.

## 2. Background

*Mycoplasma hyopneumoniae* is the aetiological agent of porcine enzootic pneumonia, an economically significant disease afflicting swine production globally [1]. Like other members of the class Mollicutes, *M. hyopneumoniae* evolved by a process of reductive evolution from the low G + C Firmicutes. The genome of *M. hyopneumoniae* is small (893–920 kb) and lacks the genetic repertoire to construct a cell wall or perform oxidative phosphorylation via the TCA cycle, and is reliant on swine for the availability of macromolecular building blocks to assemble proteins, nucleic acids and lipid membranes for growth [2,3]. As such, *M. hyopneumoniae* is armed with enzymes that degrade nucleic acids and proteins and membrane-associated transporters that facilitate uptake of the products of these degradative processes [2–4].

*M. hyopneumoniae* adheres tightly to cilia on the mucosal epithelial lining of trachea, bronchi and bronchioles of the upper respiratory tract causing ciliostasis and epithelial cell death, but the mechanism(s) deployed to destroy mucociliary function are poorly understood. Adherence is largely mediated via interactions between members of the P97 and P102 adhesin families and P159 with extracellular matrix components, glycosaminoglycans (GAGs) and fibronectin that decorate the surface of eukaryotic cells [5–9]. Members of the P97 and P102 adhesin families and P159 are large-mass (more than 100 kDa), modular, multifunctional molecules that are cleaved at multiple sites generating a complex mixture of cleavage fragments that remain non-covalently attached on the extracellular membrane surface of *M. hyopneumoniae*. Cleavage fragments derived from these adhesins bind heparin [10], fibronectin [5–7,11] and plasminogen [6,7,11,12]. The proteases responsible for cleaving the P97-family, P102-family and P159 adhesins remain unknown; however, precise cleavage sites have been determined. Efficient cleavage has been shown to occur at S/T-X-F↓X-D/E motifs found in most of the P97 and P102 adhesin families and in P159 [9,12–15]. Other efficient cleavage sites have been mapped in P97 at T-N-T↓N-T-N [16], in P159 at L-K-V↓G-A-A [14] and in P97 paralogue Mhp385 at L-N-V↓A-V-S [9]. Trypsin-like cleavage events in members of the P97 and P102 adhesin families and in P159 have also been characterized [14,15].

Recently, we showed that a dominant cleavage event occurred within a putative transmembrane domain in the N-terminus of P216 (Mhp493) with sequence ^7^T-L-L↓L↓A↓T↓A↓A↓A-I-I-G-S-T-V-F-G-T-V-V-G-L-A-S^30^ [15]. Consecutive cleavage events at positions L^10^, A^11^, T^12^, A^13^ and A^14^ are indicative of aminopeptidase activity. Aminopeptidase activity was observed at a number of putative endoproteolytic cleavage sites in Mhp493 (P216) suggesting that *M. hyopneumoniae* expresses several aminopeptidases on its cell surface [15]. Recently, we showed that the glutamyl aminopeptidase MHJ_0125 resides within *M. hyopneumoniae* cells and on the cell surface of *M. hyopneumoniae* [4]. MHJ_0125 efficiently cleaved glutamic acid, alanine and leucine but not aspartic acid, proline, valine, phenylalanine or arginine, consistent with it being classified as a member of the M42 glutamyl aminopeptidase family. Additionally, we showed that MHJ_0125 binds to porcine plasminogen and the interaction facilitates cleavage of plasminogen to plasmin by tissue plasminogen activator (tPA) [4]. Plasmin is increasingly recognized to play a key role as a proinflammatory agonist [17,18]. We observed elevated levels of plasmin and proinflammatory cytokines in bronchial fluids of pigs experimentally infected with *M. hyopneumoniae* [7] but not in experimentally infected pigs vaccinated with a commercial bacterin formulation [19]. Notably, we observed a positive correlation with bacterial load and plasmin levels indicating that *M. hyopneumoniae* is able to proliferate in the porcine respiratory tract during an inflammatory response. Plasmin is known to initiate a proteolytic cascade by activating matrix metalloproteases that cleave extracellular matrix and other circulatory host molecules, generating neo-N-terminal substrates for extracellular aminopeptidases [4].

Genome-reduced pathogens that rely heavily on their host for the supply of essential metabolic precursors are likely to benefit from increased plasmin activity at the site of infection. Our proteome studies identified a putative leucine aminopeptidase (LAP; MHJ_0461) to be exposed on the cell surface of *M. hyopneumoniae*. Like MHJ_0125, MHJ_0461 lacks evidence of a signal sequence or stretches of hydrophobic amino acids sufficient to traverse the cell membrane, and both are predicted by PSORTb to reside in the cytosol of *M. hyopneumoniae*. LAPs are proteases with broad substrate specificity but preferentially cleave N-terminal leucine residues [20]. In addition to a primary role in nutrient acquisition, microbial LAPs have been shown to bind DNA, mediate site-specific recombination and transcriptional control in *Escherichia coli* [21], regulate hydrogen sulfide production [22], activate toxins [23] and contribute to biofilm formation [24]. Here, we expressed and purified recombinant MHJ_0461 (rMHJ_0461) as a polyhistidine fusion protein, determined its substrate specificity, preference for metal ion cofactors and optimal pH range, and used comparative modelling to predict its three-dimensional structure. Bioinformatic analyses indicated that LAP carries a number of putative heparin-binding motifs. A series of binding assays were used to determine the validity of these putative binding functions.

## 3. Material and methods

### 3.1. Materials

Aminomethylcoumarin (AMC)-coupled amino acid substrates were purchased from both Bachem (UK) and Peptide Institute, Inc. (Japan). Amastatin, bestatin, ethylenediaminetetraacetic acid (EDTA), tributylphosphine (TBP), insulin, heparin, substance P, bovine serum albumin (BSA), streptavidin-peroxidase and 3, 3-diaminobenzidine were purchased from Sigma (Australia). MS grade trypsin was purchased from Promega (USA). Acrylamide was purchased from Bio-Rad (USA). Pre-cast gels, buffers, molecular weight markers and all standard molecular biology reagents were purchased from Life Technologies (Australia), unless otherwise noted.

### 3.2. *Mycoplasma hyopneumoniae* culture conditions

*M. hyopneumoniae* cells were grown in modified Friis media [25] for 48 h at 37°C while rolling. Cells were pelleted by centrifugation at 12 000×*g* for 15 min and stored at −80°C until use.

### 3.3. Expression and purification of rMHJ_0461

The *mhj_0461* gene was synthesized and cloned into the expression vector PS100030 by Blue Heron Biotech (USA) removing in frame TGA codons. In mycoplasmas, the TGA codon encodes for tryptophan, which results in truncated proteins when expressing *Mycoplasma* genes in *E. coli* [26]. In frame TGA codons were mutagenized to TGG (sequence in the electronic supplementary material) and the recombinant construct was transformed into BL21 (Invitrogen, USA) using standard protocols outlined in the manufacturer's instructions. Polyhistidine tagged rMHJ_0461 was purified under native conditions using 50% slurry of Profinity immobilized metal affinity chromatography Ni^2+^-charged resin (Bio-Rad) as per the manufacturer's instructions. Briefly, a cleared BL21 cell lysate was mixed with Ni^2+^ resin overnight at 4°C, loaded into a 10 ml column and washed twice with 4 ml wash buffer (50 mM NaH_2_PO_4_, 300 mM NaCl, 20 mM imidazole, pH 8). Bound proteins were eluted in elution buffer (50 mM NaH_2_PO_4_, 300 mM NaCl, 250 mM imidazole, pH 8), dialysed against PBS in 10 K MWCO dialysis tubing and stored at either 4°C or −20°C.

### 3.4. SDS-PAGE

Protein samples were prepared by adding 10 mM TBP and boiling at 99°C in SDS sample buffer (50 mM Tris-HCl, 2% SDS, 10% glycerol, 0.02% bromophenol blue) for 10 min. Proteins were separated by SDS-PAGE, stained overnight in Coomassie blue G250 and destained in 1% acetic acid as described previously [5]. Samples separated by native PAGE were prepared and run as described previously [27].

### 3.5. Proteomics

Details regarding peptide preparation for MS analysis have been described previously [13]. Briefly, gel bands stained with Coomassie blue were destained with 50% acetonitrile in 50 mM NH_4_HCO_3_ and then reduced and alkylated in 5 mM TBP, 20 mM acrylamide and 10 mM NH_4_HCO_3_. Each gel piece was incubated overnight at 37°C with 12.5 ng µl^−1^ Trypsin Gold MS grade (Promega) and the tryptic peptides were solubilized with 2% formic acid (v/v). Peptide samples were analysed using a TEMPO nanoLC system (Eksigent, USA) coupled to a QSTAR Elite Quadrupole TOF MS (Applied Biosystems/MDS Sciex). Intelligent Data Acquisition was performed to analyse charged ions (2+ to 5+) that were detected at greater than 30 counts per scan. MS/MS data files were searched usingMascotDaemon (v. 2.3.02) against the LudwigNR database using the following parameters: fixed modifications: none; variable modifications: propionamide, oxidized methionine, deamidated asparagine and glutamine; enzyme: semitrypsin; number of allowed missed cleavages: 3; peptide mass tolerance: 100 ppm; MS/MS mass tolerance: 0.2 Da; charge state: 2+, 3+ and 4+. The results of the search were then filtered by including only protein hits with at least one unique peptide and excluding proteins identified by a single peptide hit with a *p*-value > 0.05.

### 3.6. Enzymatic activity assays

To determine the specificities of N-terminal amino acid cleavage, 30 nM rMHJ_0461 or freshly cultured *M. hyopneumoniae* cells were added to 50 µM AMC-coupled substrates in combination with 5 mM metal cofactors (ZnCl_2_, CaCl_2_, CuCl_2_, MnCl_2_, MgCl_2_ and CoCl_2_) and a range of pH conditions (50 mM of either sodium acetate (pH 4–5.5), Tris-HCl (pH 6–8.8) or sodium borate (pH 10)). For inhibition studies, prior to the addition of substrate, rMHJ_0461 or *M. hyopneumoniae* cells were incubated with 1 mM bestatin or 1 mM amastatin for 20 min. For all assays, fluorescence was measured using a 96-well ELISA plate with a Synergy HT multi-mode microplate reader (BioTek, USA) linked to gen5 v. 1.08 software (BioTek). Reactions were mixed for 2 s immediately prior to fluorometric analysis. Assays were every 60 s for 1 h at a wavelength of 360 nm and 460/40 nm at 37°C.

### 3.7. Computational modelling and bioinformatics

Comparative molecular modelling of MHJ_0461 was performed using modeller [28]. The most suitable template for three-dimensional structure construction was obtained through BLAST search and the align2d function of modeller. Catalytic sites were deduced from sequence searches by NCBI Sequence Viewer v. 2.21 and alignments to both prokaryotes and eukaryotes using EMBL-EBI Clustal Omega. Solvent accessibility of MHJ_0461 lysine residues were predicted using PHDacc [29]. Consensus and putative heparin-binding sites were searched using ScanProsite [30] and inputted manually into modeller. DNA-binding motifs were searched using GYM 2.0 [31] and DNA-binding amino acid residues were identified using BindN [32]. Final LAP structures were rendered using the Chimera molecular modelling system v. 1.8.1 [33]. Cell localization, transmembrane domain and signal peptide predictions were made using PSORTb [34], TMpred [35] and SignalP v. 4.0 [36], respectively.

### 3.8. Antisera generation

Antisera against rMHJ_0461 were generated using New Zealand White rabbits following a protocol described previously [37].

### 3.9. Immunofluorescence microscopy

Microscopy was performed following the same protocol as in [4]. Briefly, 1 ml of *M. hyopneumoniae* strain J culture was centrifuged at 10 000×*g* for 10 min and washed three times with 1 ml sterile PBS. A 1 in 100 dilution of cells was made in PBS and added to glass coverslips and allowed to settle for 15 min at room temperature. Paraformaldehyde (4%) was added and incubated at room temperature for 30 min. Non-specific binding sites were blocked using 2% BSA in PBS overnight at 4°C. Cells were incubated with either a 1 in 100 dilution of rMHJ_0461 antisera or control rabbit sera for 1 h at room temperature, followed by 1 h incubation at room temperature with 1 in 1000 dilution of goat anti-rabbit antibodies conjugated to Alexa Fluor 488 (Life Technologies). Control sera were collected from rabbits prior to immunization with rMHJ_0461. Coverslips were mounted in VECTASHIELD onto microscope slides and imaged using an Olympus BX51 Upright Epi Fluorescence microscope. Images were captured using an Olympus DP97 Digital Microscope Camera coupled with Olympus DP Controller software.

### 3.10. Cell surface analyses of *Mycoplasma hyopneumoniae*

*M. hyopneumoniae* surfaceome analysis was performed using both cell surface biotinylation and enzymatic cell surface shaving with trypsin as previously described [11,12].

### 3.11. Heparin binding chromatography and microscale thermophoresis

Heparin affinity chromatography was performed using Waters 2690 Alliance LC separations modules as described previously [12]. Briefly, *M. hyopneumoniae* whole cell lysates were run through 1 ml HiTrap Heparin HP columns (GE Healthcare, Australia) and fractions collected and separated into low and high affinity interactions in accordance to an elution profile based on an increasing salt gradient. These fractions were then separated by SDS-PAGE and proteins were identified by LC-MS/MS.

For microscale thermophoresis (MST) analysis of binding kinetics between fluorescently labelled heparin and rMHJ_0461, samples were prepared as per manufacturer's instructions. Briefly, 20 μl of rMHJ_0461 at a concentration of 5 μM was added to a reaction tube. Ten microlitres of PBS with 0.05% (v/v) Tween 20 was then added to an additional 15 reaction tubes. Serial dilutions were made by transferring 10 μl from the first tube to the next, discarding 10 μl from the last tube after transfer. Ten microlitres of 2 μM heparin labelled with red fluorescent dye NT-647 (NanoTemper) was added to each tube and incubated at room temperature for 1 h. Samples were then loaded into hydrophilic capillaries (NanoTemper) and MST was executed on a NanoTemper Mononlith NT.115 using the following parameters at 24°C: LED power set at 50%, MST power at 40, 60 and 80% with fluorescence measurements taken after 30 s. All experiments were performed in triplicate.

### 3.12. Porcine plasminogen activation assay

The ability of rMHJ_0461 to influence the conversion of plasminogen to plasmin in the presence of tPA was determined using a method described previously [7]. Purified porcine plasminogen (50 μg ml^−1^) [11] was incubated for 1 h at 37°C with 1 : 0.5, 1 : 1, 1 : 2, 1 : 4 and 1 : 8 molar ratios of rMHJ_0461 in microtitre plate wells (Greiner Bio One, Germany) before the addition of tPA and Spectrozyme-PL. Controls included plasminogen alone, plasminogen and rMHJ_0461 in the absence of tPA, plasminogen and tPA in the absence of rMHJ_0461. Protein controls using substance P and insulin as substitutes for rMHJ_0461 at 1 : 1 molar ratios were also tested. Absorbance at 405 nm was read every 5 min for 90 min. This experiment was performed twice, each time in triplicate.

### 3.13. Ligand blot analysis

Ligand blotting was used to determine whether rMHJ_0461 binds plasminogen. Serially diluted rMHJ_0461 was spotted on three PBS soaked nitrocellulose Hybond C-Super membranes assembled in a Bio-Dot Microfiltration apparatus and the wells were washed three times with 50 μl of PBS under gravity filtration. Membranes were blocked with 1% skim milk powder in PBS Tween 20 (0.1% v/v). Two membranes were then incubated with a 1 in 1000 dilution of biotinylated plasminogen prepared as described previously [4] for 90 min at room temperature. One blot was incubated in the presence of 1 M *ɛ*-aminocaproic acid. The remaining membrane was used as a control and incubated in PBS only. All three membranes were then probed with streptavidin-peroxidase (1 in 3000 dilution) for 60 min and developed using 3, 3-diaminobenzidine peroxidase substrate.

### 3.14. DNA-binding assay

Salmon sperm DNA (Sigma D1626) was dissolved in nuclease-free water to make a solution at 2 mg ml^−1^. DNA fragments (800 bp) of salmon sperm DNA were generated by sonicating 130 μl of 1 mg ml^−1^ DNA solution in a Covaris M220 for 80 s (peak power at 50, duty factor of 5.0, for 200 cycles). Protein–DNA binding preparations were set up as described previously [38], incubating 0.1 mg ml^−1^ of DNA with approximately 1 mg ml^−1^ of rMHJ_0461. All assays were completed in DNA-binding buffer (10 mM Tris-HCl, 125 mM KCl, 10 mM MgCl_2_, 0.1 mM dithiothreitol, 5% glycerol, pH 7.2) for 30 min at 37°C. DNA : protein dilutions 1 : 1, 1 : 10, 1 : 20, 1 : 50, 1 : 100, BSA and DNA controls were run on a Bioanalyzer 2100 High Sensitivity chip in the Agilent 2100 Bioanalyzer as per the manufacturer's instructions. Briefly, gel–dye mix was prepared to allow high sensitivity DNA dye concentrate (blue) and high sensitivity DNA gel matrix (red) to equilibrate to room temperature for 30 min. Fifteen microlitres of high sensitivity DNA dye concentrate (blue) was added to a high sensitivity DNA gel matrix vial (red), vortexed, pulse spun for 5 s and transferred to a spin filter to be centrifuged at 2240×*g* ± 20% for 10 min. Using a new high sensitivity DNA chip on the chip priming station, 9.0 µl of gel–dye mix was added in wells marked ‘G’ and 5 µl of marker (green) was added into all sample and ladder wells. One microlitre of high sensitivity DNA ladder (yellow) was used. The chip was placed horizontally in the adapter and vortexed for 1 min at 2400 r.p.m. The chip was then run in the Agilent 2100 Bioanalyzer using standard high sensitivity settings.

## 4. Results

### 4.1. Localization of MHJ_0461 in *Mycoplasma hyopneumoniae*

**Figure 1.**
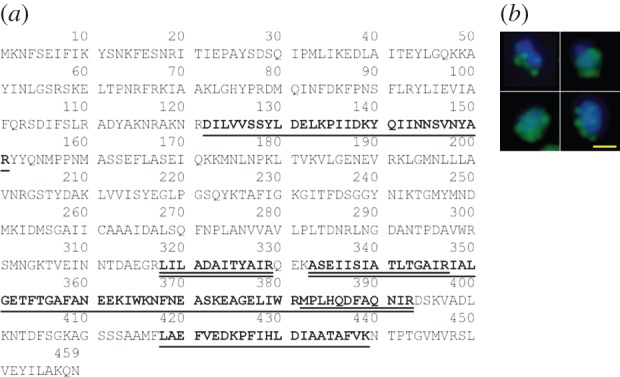
LAP is present on the surface of *M. hyopneumoniae* cells. (*a*) Underlined: tryptic peptides released by mild trypsin digestion of cell surface proteins that map to MHJ_0461. Double underlined: tryptic peptides mapping to MHJ_0461 that were identified after biotinylated surface proteins were recovered by avidin chromatography and digested with trypsin. (*b*) An overlayed image illustrating surface localization of MHJ_0461. Whole *M. hyopneumoniae* cells were probed with rabbit anti-MHJ_0461 serum and anti-rabbit antibodies conjugated to Alexa Fluor 488 (green). *M. hyopneumoniae* cells were also stained with the nucleic acid stain DAPI (blue). Yellow bar represents 5 μm.

MHJ_0461 has a predicted mass and pI of 51.4 kDa and 8.85, respectively. Cell surface shaving experiments using viable, freshly cultured *M. hyopneumoniae* cells showed tryptic peptides mapping to MHJ_0461 (figure 1*a*) were detected by LC-MS/MS, indicating that MHJ_0461 resides on the cell surface. To confirm the surface accessibility of MHJ_0461, freshly cultured *M. hyopneumoniae* cells were briefly labelled with biotin and biotinylated proteins were recovered by avidin chromatography, separated by SDS-PAGE and subjected to LC-MS/MS. Tryptic peptides that mapped to MHJ_0461 were detected using this approach, confirming that MHJ_0461 resides on the cell surface of *M. hyopneumoniae* (figure 1*a*). These techniques have been used extensively to reliably determine the cell surface location of other proteins in *M. hyopneumoniae* [4,16]. We further confirmed that MHJ_0461 is bound to the surface of *M. hyopneumoniae* by immunofluorescence microscopy using mono-specific polyclonal antibodies raised against rMHJ_0461. Bound antibodies were detected on the surface of freshly grown *M. hyopneumoniae* cells with anti-rabbit antibodies conjugated with Alexa Fluor 488. In control experiments, antibodies in rabbit antiserum collected from the same rabbit but prior to immunization did not bind to the surface of *M. hyopneumoniae* cells (data not shown). All *M. hyopneumoniae* cells that stained with the nuclear stain 4′, 6-diamidino-2-phenylindole (DAPI) displayed MHJ_0461 on the cell surface (examples seen in figure 1*b*).

### 4.2. Biochemical studies with enzymatically active rMHJ_0461

**Figure 2.**
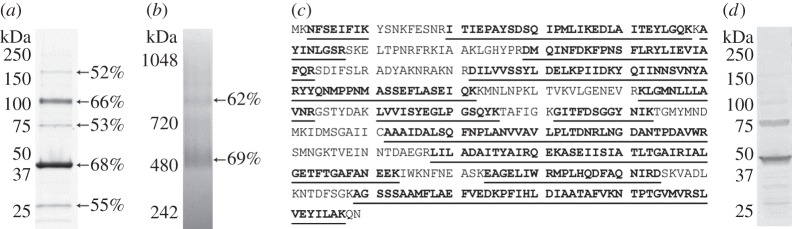
rMHJ_0461 resolves as multiple migratory forms when purified under native and denaturing conditions. (*a*) SDS-PAGE gel showing purified rMHJ_0461 resolves in multiple forms after reduction, alkylation and boiling in SDS. Detection is by Coomassie staining. (*b*) Native PAGE resolved large mass multimeric forms of rMHJ_0461. In both (*a*,*b*), percentages represent tryptic peptide coverage to rMHJ_0461 coverage as determined by LC-MS/MS. (*c*) Tryptic peptide matches (underlined) of monomeric rMHJ_0461 that migrates at approximately 47 kDa. (*d*) Western blot probed with rabbit anti-rMHJ_0461 serum demonstrating rMHJ_0461 multimers observed by SDS-PAGE are also observed when a whole cell lysate *of M. hyopneumoniae* is probed with anti-rMHJ_0461 serum.

rMHJ_0461 resolved as five bands during SDS-PAGE and the different forms of rMHJ_0461 were unaffected by conditions used for reduction and alkylation (figure 2*a*). All five migratory forms of the protein were digested with trypsin and confirmed to be rMHJ_0461 by LC-MS/MS. Tryptic peptide coverage to MHJ_0461 ranged from 52 to 68% (figure 2*a*) and our analyses did not find any tryptic peptides that mapped to contaminating *E. coli* proteins. rMHJ_0461 resolves during Blue Native PAGE as two complexes of approximately 500 and 800 kDa, suggesting that MHJ_0461 forms multimers comprising more than 10 subunits. Each complex generated tryptic peptides spanning 62% and 69% of MHJ_0461 sequence, respectively (figure 2*b*). Multimeric forms of MHJ_0461 were also evident on blots of whole cell lysates of *M. hyopneumoniae* probed with rabbit anti-rMHJ_0461 sera (figure 2*d*).

**Figure 3.**
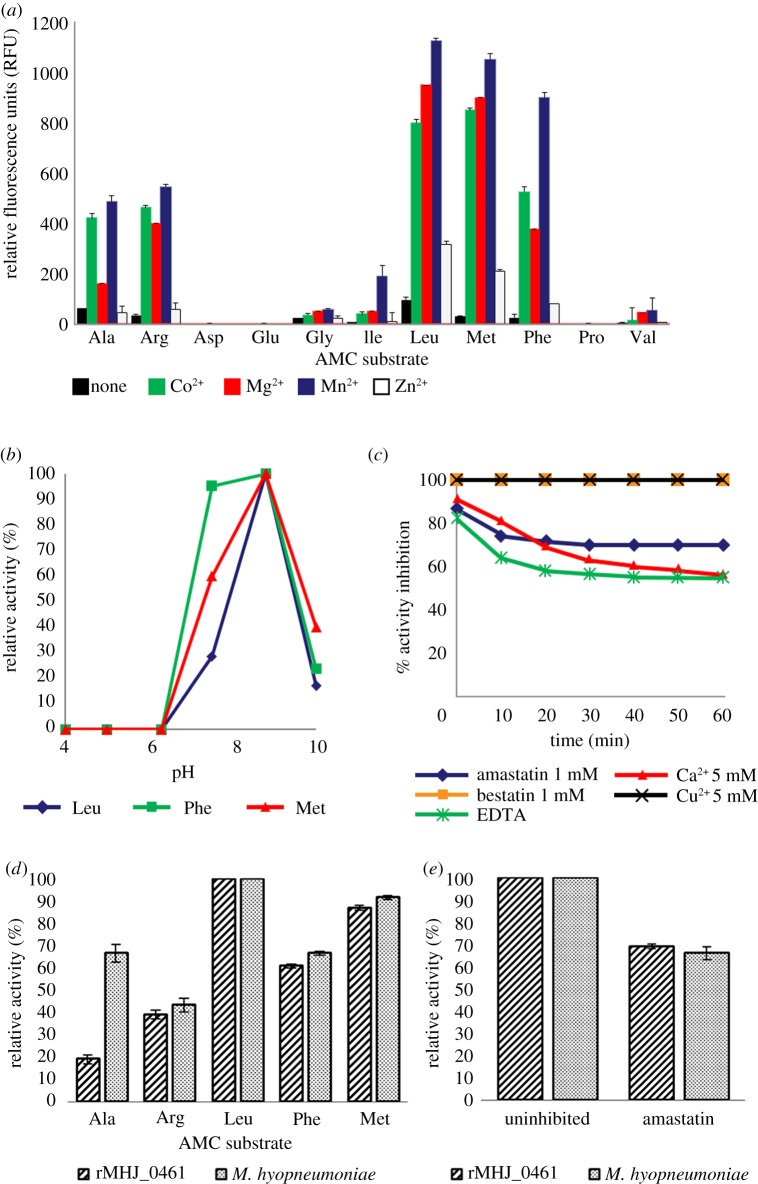
Biochemical characterization of rMHJ_0461. (*a*) N-terminal amino acid substrate specificity of rMHJ_0461 at pH 7.2 in the presence of metal cofactors. (*b*) Activity of rMHJ_0461 against leucine, methionine and phenylalanine substrates evident from pH 7.2 to pH 10. Optimal activity was observed at pH 8.8. (*c*) Activity of rMHJ_0461 against leucine-AMC in the presence of different inhibitors. Activity was inhibited 100% by bestatin and Cu^2+^, 69% by amastatin, 60% by Ca^2+^ and 58% by EDTA. (*d*) Comparative substrate activity using five AMC-coupled amino acids for rMHJ_0461 and live *M. hyopneumoniae*. (*e*) Activity against leucine-AMC by rMHJ_0461 and live *M. hyopneumoniae* cells was inhibited by 1 mM amastatin by 69% and 66%, respectively.

The specificity of rMHJ_0461 for N-terminal amino acids was determined using AMC-coupled amino acids in the presence of various divalent cations using a standard kinetic assay. At pH 7.2, rMHJ_0461 demonstrated greatest aminopeptidase activity against leucine-coupled substrates but also exhibited strong activity against other amino acids with hydrophobic side chains including methionine, phenylalanine and alanine and positively charged arginine. Weaker activity was observed with isoleucine-, glycine- and valine-coupled substrates but no activity was observed for proline and negatively charged aspartic and glutamic acids. In the absence of divalent cations, rMHJ_0461 displayed limited substrate hydrolysis. Aminopeptidase activity was strongest in the presence of Mn^2+^ in the order of leucine > methionine > phenylalanine > arginine > alanine > isoleucine > glycine > valine. Cleavage at N-terminal residues was also greatly increased in the presence of Co^2+^ and Mg^2+^, and to a lesser extent in the presence of Zn^2+^ (figure 3*a*). The binding of different metal ions can alter protein structure, referred to as a metalloform. This explains not only why activity is altered in the presence of different cofactors, but also why inhibitors of one protease metalloform may not inhibit the same protease in another metalloform [39].

LAPs are generally most active across neutral and basic pH levels but exceptions have been reported [40]. Activity of rMHJ_0461 against leucine-, methionine- and phenylalanine AMC-coupled substrate in the presence of Mn^2+^ was tested using a range of buffered substrates from pH 4 to pH 10. For each substrate, activity was not apparent in acidic conditions. Aminopeptidase activity was first observed at pH 7.2, peaked at pH 8.8 and remained detectable at pH 10 (figure 3*b*).

Aminopeptidase activity against leucine-AMC substrate in the presence of Mn^2+^ was reduced by 99% after incubation with 1 mM bestatin, 69% by 1 mM amastatin and 58% by 10 mM EDTA. Copper and calcium divalent ions at 5 mM were also found to inhibit activity by 99% and 60%, respectively (figure 3*c*). Metal inhibitory agents were explored as one current drug development strategy involves the use of metal complexes that bind to the active site of a protease, leading to competitive inhibition [41].

To determine if aminopeptidase activity is present on the surface of *M. hyopneumoniae*, freshly cultured *M. hyopneumoniae* cells were incubated with AMC-coupled substrates. With the exception of alanine-AMC, aminopeptidase activity was analogous with the rMHJ_0461 substrate specificity profile described above (figure 3*d*). Leucine-AMC activity on the cell surface was inhibited by 1 mM amastatin by 66%, a level comparable to the 69% activity reduction seen in rMHJ_0461 (figure 3*e*).

### 4.3. Comparative modelling of MHJ_0461

Comparative molecular modelling of MHJ_0461 and its catalytic sites was performed using the program modeller. LAP from *E. coli* (PDB 1GYT, sequence identity 26%) was identified as the most suitable template for modelling *M. hyopneumoniae* MHJ_0461 with a GA341 score of 1.00 indicating more than 95% probability of having the correct fold [42], and a discrete optimized protein energy (DOPE) score of −0.37. A DOPE score below −1 is likely to be native-like [43].

**Figure 4.**
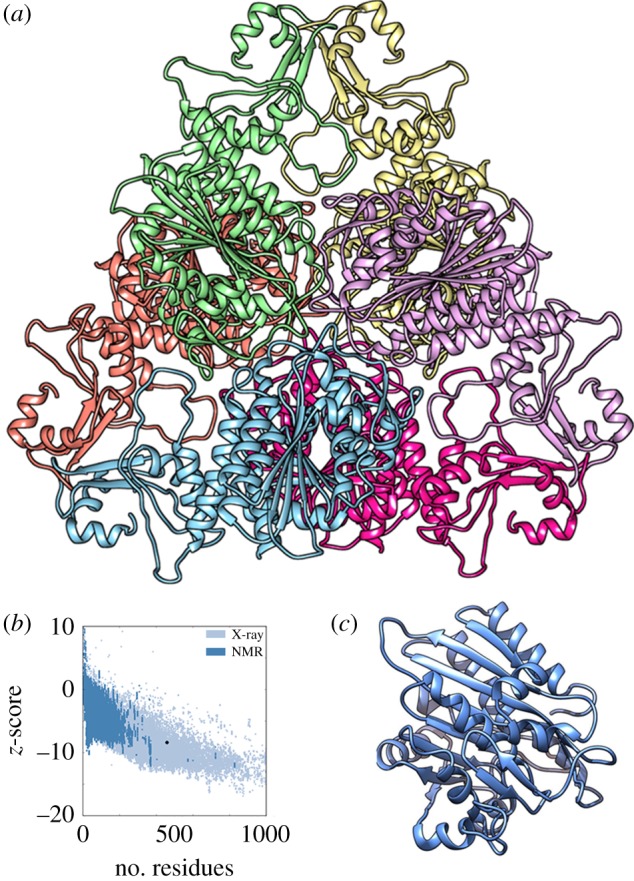
Comparative molecular modelling of MHJ_0461. (*a*) Ribbon structure of MHJ_0461 presented as a homohexamer with each subunit represented in a different colour. The template used for modelling was *E. coli* LAP PDB 1GYT. (*b*) A *Z*-score of −8.34 was determined for the model depicted in panel (*a*). (*c*) Monomeric ribbon structure of MHJ_0461.

LAPs typically display hexameric tertiary structures [20,44]. The model ribbon structure of MHJ_0461 from *M. hyopneumoniae* presented as a homohexamer is shown in figure 4*a*. ProSA assigned a *Z*-score of 8.34 with all residues falling within the midrange of available nuclear magnetic resonance and X-ray crystallography structures currently available (figure 4*b*).

**Figure 5.**
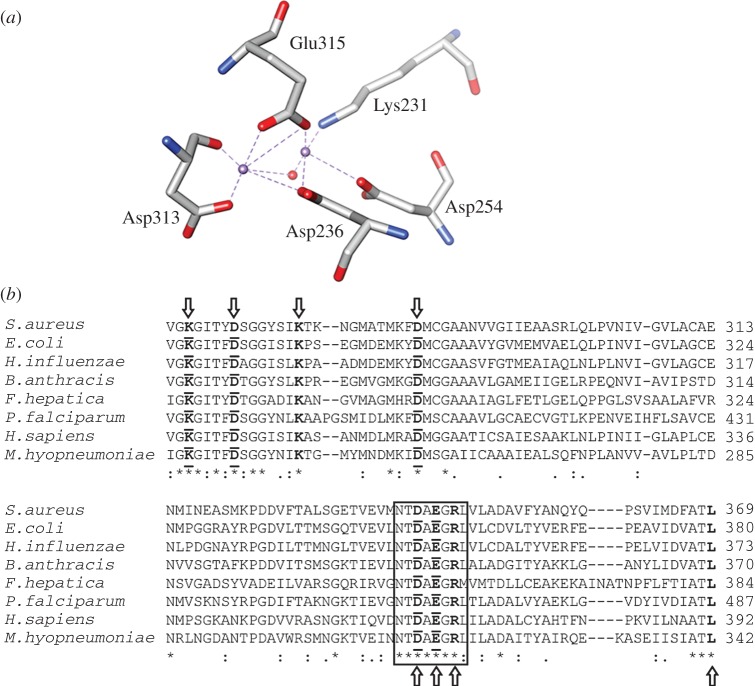
Active sites of LAPs are highly conserved. (*a*) Model metal binding site of MHJ_0461 showing two divalent cations (purple) covalently bound to three amino acid ligands each plus a water molecule (red). (*b*) Amino acid sequence alignment of Gram-positive, Gram-negative, apicomplexan and eukaryotic LAPs demonstrates low overall sequence identity but highly conserved metal (bold and underlined) and substrate (bold) binding sites. The NTDAEGRL motif characteristic of the M17 superfamily of metalloproteases is boxed.

The model catalytic site for MHJ_0461 displays two Mn^2+^ cations coordinated in trigonal prismatic geometry to ligands Lys231, Asp236, Asp254, Asp313 and Glu315. A water molecule also takes part in the nucleophilic cleavage of a substrate (figure 5*a*). MHJ_0461 from *M. hyopneumoniae* strain J shares high sequence identity with putative LAPs from different strains of *M. hyopneumoniae* (98–100%). Sequence identity is considerably lower when MHJ_0461 is aligned with putative LAPs from other *Mycoplasma* sp. including *Mycoplasma fermentans* (43%) and *Mycoplasma bovis* (42%) and orthologues from phylogenetically related bacteria including *Clostridium perfringens* (42%) and *Bacillus cereus* (38%). A low sequence identity was observed throughout the entire molecule when aligned against a variety of organisms ranging from 31% for *Bacillus anthracis* to 20% for *Fasciola hepatica*. However, the metal and substrate binding sites and the NTDEAGRL motif characteristic to M17 family proteases [20] are highly conserved across prokaryotic and eukaryotic LAPs and present in MHJ_0461 (figure 5*b*).

### 4.4. MHJ_0461 binds heparin

**Figure 6.**
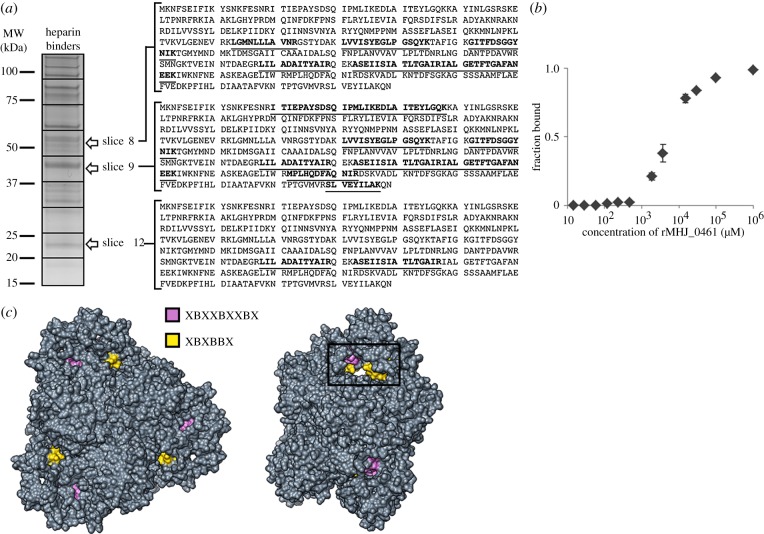
MHJ_0461 binds heparin. (*a*) rMHJ_0461 was identified in a high affinity (700 mM NaCl) heparin-binding fraction (E2) at three molecular masses by LC-MS/MS. Matched peptides are underlined. (*b*) Thermophoretic movement of rMHJ_0461 in the presence of heparin. (*c*) Two viewpoints depicting the surface topography of MHJ_0461 demonstrate that heparan sulfate-binding motif XBXBBX (yellow) and putative heparin-binding motif XBXXBXXBX (purple) are surface accessible and localized around pores (boxed).

Putative heparin-binding proteins of *M. hyopneumoniae* were identified by heparin-agarose chromatography. Soluble proteins retained during heparin-agarose chromatography were eluted with a salt gradient from 300 to 2000 mM, separated by SDS-PAGE and identified by LC-MS/MS. Peptides matching MHJ_0461 were detected in three gel slices (figure 6*a*), suggesting that different forms of the molecule retain the ability to bind heparin. MST studies showed rMHJ_0461 bound heparin with a dissociation constant (Kd) of 6.89 nM (figure 6*b*). We examined the sequence of MHJ_0461 for motifs that may play a role in binding heparin. The motif XBXBBX (where B = H, K or R and X = any amino acid) located at amino acid residues 64–69 has been implicated in binding heparan sulfate [45]. The binding site was added manually into modeller, rendered over the predicted ribbon structure and shown to be surface accessible around a large pore. An additional cluster of repetitively spaced basic residues with the sequence XBXXBXXBX was identified between amino acids 71 and 79 located around the same pore (figure 6*c*).

### 4.5. rMHJ_0461 binds plasminogen and facilitates plasmin conversion

**Figure 7.**
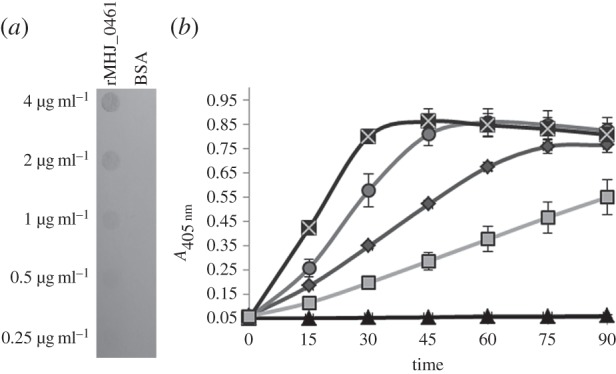
rMHJ_0461 binds plasminogen and facilitates its conversion to plasmin in the presence of tPa. (*a*) Ligand blot of rMHJ_0461 probed with porcine plasminogen. In descending order, rMHJ_0461 and BSA (negative control) were blotted in concentrations of 4, 2, 1, 0.5 and 0.25 μg ml^−1^. (*b*) In the presence of tPA, rMHJ_0461 increases plasminogen activation to plasmin. An increase in the rate of plasmin activity is evident in the presence of both 1 : 1 rMHJ_0461 to plasminogen molar ratio (circles) and 4 : 1 (crosses) in comparison to plasminogen and tPA alone (diamonds). A 1 : 1 insulin to plasminogen ratio decreased plasmin activity (squares) and no plasmin activity was observed with rMHJ_0461 and plasminogen in the absence of tPA (triangles).

Several bacterial species manipulate host defences by commandeering host plasminogen [46]. Many cell surface proteins bind plasminogen and facilitate its conversion to plasmin by enhancing the accessibility to host plasminogen activating enzymes urokinase plasminogen activator and tPA or by producing bacterial surface-associated activators that interact with plasminogen and activate it by complex formation [47]. rMHJ_0461 bound plasminogen in a concentration-dependent manner (figure 7*a*). In the presence of a lysine analogue, *ɛ*-aminocaproic acid, binding of porcine plasminogen to rMHJ_0461 was diminished, suggesting surface-exposed lysines are critical in binding interactions (western blot in electronic supplementary material). In the presence of tPA, there was a distinct increase in plasmin activity (figure 7*b*) at all molar ratios of rMHJ_0461 to plasminogen, ranging from 0.5 : 1 to 8 : 1. In control experiments using plasminogen and tPA, maximum plasmin activity was reached by 75 min. The same level of activity was reached with an incubation time of 60 min when rMHJ_0461 was present at a 0.5 : 1 molar ratio, by 35 min at 1 : 1 and 2 : 1 molar ratios and by 20 min at molar excess ratios 4 : 1 and 8 : 1. To examine the role of molecular crowding, we performed control experiments by measuring plasmin activity when plasminogen was incubated with substance P and insulin. In both instances, plasmin activity was lower and did not reach the same level of absorbance by 90 min in the presence of substance P and insulin at 1 : 1 molar ratios. No plasmin activity was observed when rMHJ_0461 was incubated with plasminogen in the absence of tPA, indicating that MHJ_0461 is incapable of directly cleaving plasminogen to release plasmin.

### 4.6. rMHJ_0461 interacts with DNA

**Figure 8.**
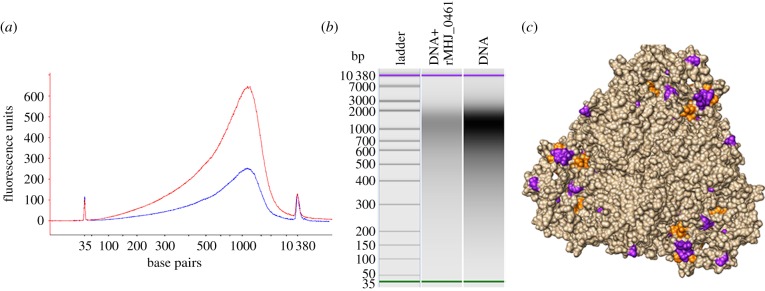
rMHJ_0461 binds DNA. (*a*) Electropherogram showing relative fluorescence of DNA (red) and rMHJ_0461 : DNA (blue) samples. (*b*) Bioanalyzer gel image with visibly lower concentration of DNA detected in rMHJ_0461 : DNA sample. Starting DNA concentration was 1231.28 pmol l^−1^. Amount of DNA detected in rMHJ_0461 : DNA sample was 445.07 pmol l^−1^ suggesting rMHJ_0461 bound 64% of the sample DNA. (*c*) Putative HTH motif (orange) and DNA-binding residues (purple) are depicted on the surface topology of the MHJ_0461 homohexamer.

LAP from *E. coli* (PDB 1GYT) was selected as the most statistically suitable template to model a proposed three-dimensional structure for MHJ_0461. *E. coli* LAP is known to bind DNA, and the interaction with DNA is suggested to be structural as there are no known DNA-binding motifs in the sequence [21]. To investigate whether rMHJ_0461 binds DNA, the protein was incubated with salmon sperm DNA at a 1 : 1 ratio at 37°C for an hour and run on a Bioanalyzer to detect changes to the concentration of the DNA. In the presence of rMHJ_0461, the concentration of DNA decreased by approximately 64% (figure 8*a*,*b*). In control experiments where DNA was incubated with BSA, no change in DNA concentration was observed as expected (data not shown). Bioinformatic analysis of rMHJ_0461 identified one helix-turn-helix (HTH) motif common in DNA-binding proteins spanning amino acids 66–88. Additionally, 18 DNA-binding residues were identified using the BindN algorithm [32], 65% of which were located within the N-terminal region of MHJ_0461. These motifs and residues are concentrated at the outer corners of hexameric MHJ_0461 (figure 8*c*).

## 5. Discussion

Aminopeptidases have long been known to play an important role in nutrient acquisition and cell homeostasis. However, it is also becoming apparent that aminopeptidases are multifunctional proteins with secondary functions (i) as viral or toxin receptors, (ii) as site-specific recombination factors, (iii) as transcriptional repressors and (iv) in vesicular trafficking [20]. These functions indicate that aminopeptidases can reside in multiple cellular compartments. While many of these moonlighting functions have been described in eukaryote systems, it is now clear that aminopeptideases are multifunctional proteins in prokaryotes. Here, we show that MHJ_0461 resides in different cellular locations where it functions as a LAP with broad substrate specificity. MHJ_0461 does not possess an N-terminal transmembrane domain and is predicted by PSORTb to have a cytosolic location. However, tryspin shaving and surface biotinylation studies identified MHJ_0461 on the surface of *M. hyopneumoniae*, an observation confirmed by immunofluorescence microscopy using anti-MHJ_0461 antibodies. MHJ_0461 also displays additional moonlighting functions by binding heparin, plasminogen and DNA, indicating that MHJ_0461 plays an important role in survival of *M. hyopneumoniae* in the host and in pathogenesis more broadly.

Activity against a range of fluorogenic substrates was detected in freshly cultured *M. hyopneumoniae* cells (figure 3*d*). With the exception of alanine, the activity profiles were comparable with the substrate specificity profile of rMHJ_0461 for the panel of amino acids tested. The additional alanine activity could be attributed to surface-exposed glutamyl aminopeptidase (MHJ_0125) previously shown to have high activity against alanine-AMC [4]. Leucine-AMC activity was inhibited by amastatin by 66%, which was analogous to the 69% activity reduction seen in rMHJ_0461. These results indicate that MHJ_0461 retains its aminopeptidase activity on the surface of *M. hyopneumoniae*. LAP activity has been described on the surface of *Mycoplasma bovirhinis*, *Mycoplasma dispar* and *M. bovis* [48]. Genome sequences for *M. bovirhinis* and *M. dispar* are not available; however, three strains of *M. bovis* have been sequenced revealing five predicted cytosolic aminopeptidases. Like MHJ_0461, none of these putative aminopeptidases possess signal peptides. These data suggest that active aminopeptidases from pathogenic mycoplasmas are transported to the cell surface albeit by an unknown mechanism.

rMHJ_0461 showed the greatest activity against leucine-AMC but also efficiently cleaved large hydrophobic residues such as methionine and phenylalanine. rMHJ_0461 substrate specificity was leucine > methionine > phenylalanine, which is comparable to LAPs from *Pseudomonas aeruginosa*, *Salmonella enterica* serotype Typhimurium, *E. coli* and *Vibrio proteolyticus* [49]. rMHJ_0461 has 80% activity against phenylalanine-AMC relative to leucine-AMC, while LAP from *Streptomyces septatus* and *V. proteolyticus* has 23% and 18% activity, respectively [50,51]. Recently, we showed that endoproteolytic fragments of P216 become targets of aminopeptidase-like activity. Cleavage fragments were detected that showed evidence of sequential loss of small (leucine and alanine) and large (tyrosine, phenylalanine) hydrophobic and polar (serine and threonine) amino acids [15]. rMHJ_0461 also efficiently cleaves the positively charged amino acid arginine, a characteristic shared with LAPs isolated from *Geobacillus thermoleovorans* [52] and *Bacillus subtilis* [53]. Notably, small N-terminal amino acid residues are considered to be poor substrates for most LAPs [51,53]. rMHJ_0461 had 40% activity against alanine-AMC compared with leucine-AMC. rMHJ_0461 was unable to hydrolyse bonds formed by negatively charged amino acids or proline in the P1′ position. Notably, rMHJ_0461 was able to cleave methionine. Consistent with other studies, our surface studies indicated that methionine aminopeptidase (MHJ_0169) is not located on the cell surface but resides intracellularly. The ability of rMHJ_0461 to cleave methionine provides *M. hyopneumoniae* with a source of this important amino acid for protein synthesis. We recently showed that the glutamyl aminopeptidase MHJ_0125 is presented on the cell surface of *M. hyopneumoniae* and was able to cleave glutamic acid, alanine and leucine but poorly hydrolysed aspartic acid, arginine, proline, valine and phenylalanine [4]. The cell surface location of both MHJ_0125 and MHJ_0461 provides *M. hyopneumoniae* with the capacity to cleave a wide range of amino acids from neo-N-termini.

MHJ_0461 can be classified as belonging to the M17 family of proteases by the presence of the NTDAEGRL motif and a C-terminal catalytic domain with highly conserved metal binding sites. These binding residues are coordinated to two divalent ions which act as positively charged electrophilic catalysts that complex an oxygen atom at a scissile peptide bond, thus facilitating the nucleophilic attack of an additionally coordinated water molecule leading to peptide bond cleavage [54]. Our data show that greatest activity across all substrates tested was achieved with the addition of manganese cations and was also increased considerably in the presence of cobalt and magnesium. The activity rMHJ_0461 was inhibited by the aminopeptidase inhibitors bestatin and amastatin and the metal chelating agent EDTA.

Adherence of *M. hyopneumoniae* to mucosal cells and respiratory cilia constitutes the first crucial stage of infection and is facilitated by surface-exposed adhesins that, in part, bind to extracellular matrix components such as fibronectin and GAGs [4–7,37]. Bioinformatic analyses identified several regions enriched in basic amino acids, suggesting that MHJ_0461 may display the ability to bind GAGs. The ability to bind respiratory tract cilia and porcine epithelial-like cells is largely abolished when *M. hyopneumoniae* is pre-incubated with the GAG heparin [10,55]. Many of the endoproteolytic cleavage fragments of the P97 and P102 adhesin families and other surface molecules bind heparin, underscoring the important role this interaction plays in the biology of this pathogen [4,5,8–12,14,37]. In this study, MHJ_0461 was shown to bind heparin by both heparin affinity chromatography and thermophoresis. We identified a heparin-binding site at amino acids 64–69 implicated in heparin sulfate binding and another highly basic cluster at amino acids 71–79, both of which were shown via molecular modelling to be situated around the largest surface pocket. As the largest pocket is where natural substrates and cofactors bind for 70–85% of enzymes [56], we propose that MHJ_0461 binds GAGs and plays a role in the processing of GAGs. These proteolytic processes may contribute to the extensive cilia and epithelial cell damage seen in chronic *M. hyopneumoniae* infections [57]. GAG binding may have even greater pathological implications as it has been linked to microbial intracellular survival within macrophages [58] and host cell invasion and dissemination [59]. Despite being considered a strictly respiratory pathogen, *M. hyopneumoniae* has been isolated from other organs, including the liver, spleen and kidney [60] as well as the brain [61]. GAG binding may facilitate cell invasion and enable *M. hyopneumoniae* to proliferate in tissue sites distal to the respiratory tract.

*M. hyopneumoniae* is very adept at recruiting plasmin(ogen) onto its cell surface and facilitating activation to plasmin. Endoproteolytic fragments of members of the P97 and P102 adhesin families bind plasminogen and promote conversion of plasminogen to plasmin in the presence of tPA [6,7,11,12]. The bronchoalveolar lavage (BAL) fluid of pigs infected experimentally with *M. hyopneumoniae* consistently showed greater plasmin activity compared with BAL fluid recovered from the same animals prior to experimental challenge. These observations suggest that elevated plasmin levels in BAL fluid are a consequence of *M. hyopneumoniae* infection [7]. This is significant for several reasons. First, plasmin is known to regulate inflammatory responses, including macrophage signalling via mitogen-activated protein kinase and nuclear factor kappa B (NF-κB) pathways and cytokine release [17,18]. Second, plasmin induces neutrophil aggregation [62], stimulates peripheral monocytes to release proinflammatory factors and recruits monocytes to the site of inflammation [63–65]. Third, several bacterial species have been shown to commandeer plasminogen on their surface to escape fibrin clot confinement and immunological clearance [46,66]. Lastly, plasmin activity is ameliorated in pigs vaccinated with bacterin formulations [19], which are known to reduce lung lesion pathology [67]. This observation suggests that plasmin plays a key role in generating the pathology induced by infections caused by *M. hyopneumoniae*. Here, we show that rMHJ_0461, like rMHJ_0125, binds plasminogen. Plasminogen bound to rMHJ_0461 was efficiently converted to plasmin in the presence of tPA. rMHJ_0461 enhanced plasmin activity at all molar ratios tested. The increase in the rate of activation was greater than that observed for rMHJ_0125 which induced plasmin conversion only in protein to plasminogen molar ratios greater than 4 : 1. The presence of control proteins substance P and insulin lowered the rate of plasmin conversion. These data suggest that the increase in activity seen in rMHJ_0461 was not due to molecular crowding and that the presence of non-binding proteins at 1 : 1 molar ratios inhibits the conversion of plasminogen to plasmin. The observation that rMHJ_0461 in the absence of tPA did not produce plasmin activity shows that the protease is unable to directly cleave the plasminogen R^561^–V^562^ bond to form plasmin consistent with it functioning as an aminopeptidase. Interactions between rMHJ_0461 and plasminogen were abolished in the presence of a lysine analogue, *ε*-aminocaproic acid, indicating that rMHJ_0461 relies on surface-exposed lysine residues for plasminogen binding.

Plasmin is an endoprotease with broad substrate specificity. Plasmin cleaves extracellular matrix proteins and plays a key role in the processing and activation of matrix metalloproteases [4]. Collectively, these events generate cleavage fragments that provide a pool of free N-termini that are substrates for aminopeptidases. MHJ_0125 and MHJ_0461 are effective at removing a wide spectrum of N-terminal amino acids. As a genome-reduced pathogen, *M. hyopneumoniae* would benefit from the pool of free amino acids generated by both these aminopeptidases and is consistent with a model we proposed earlier [4]. Metabolome studies in the human respiratory pathogen *Mycoplasma pneumoniae*, an organism also reliant on the host to provide a pool of free amino acids for growth, suggested that 354 amino acids per second must be imported into each cell during exponential growth [68]. These studies suggest that aminopeptidases are an important source of free amino acids during infection.

rMHJ_0461 was shown to bind double-stranded DNA. While further studies are required to determine the biological significance of this interaction, a putative DNA-binding motif and putative DNA-binding residues within MHJ_0461 reside within the N-terminal 100 amino acids. This observation is consistent with reports that the N-terminus of some LAPs plays an important role in binding DNA [21]. LAPs from *E. coli* have DNA-binding capabilities [21,69] which enable them to function as regulators of site-specific recombination and transcription [21]. However, these LAPs are intracellular proteins. To our knowledge LAP from *M. hyopneumoniae* is the first example of a cell surface aminopeptidase that binds DNA. Few extracellular DNA-binding proteins have been identified to date; examples include proteins involved in preventing adaptive immune responses [70] and those that form complexes with extracellular DNA and contribute to biofilm formation [71]. The role of MHJ_0461 in the pathogenesis of *M. hyopneumoniae* will be the subject of future studies.

## Supplementary Material

rMHJ_0461 sequence manipulations

## Supplementary Material

Peptide coverage for rMHJ_0461 multimeric bands

## Supplementary Material

rMHJ_0461 does not bind plasminogen in the presence of a lysine analogue
