# Supplementary material for: MHJ_0461 is a multifunctional leucine aminopeptidase on the surface of Mycoplasma hyopneumoniae
Source: Open Biol. 2015 Jan 14;5(1):140175. doi: 10.1098/rsob.140175 (PMC4313372; doi:10.1098/rsob.140175)
Supplement: rMHJ_0461 sequence manipulations [file rsob140175supp1.pdf]

mhj_0461 sequence highlighting inframe TGA codons that were changed to TGG during commercial gene synthesis.

1 ATT TTT ATC AAG TAT TCA AAT AAA TTT GAA TCC AAT CGA ATA ACC ATT

49 GAA CCA GCT TAT TCT GAT TCG CAA ATT CCA ATG TTG ATA AAA GAA GAT

97 TTA GCT ATT ACC GAG TAT CTT GGG CAA AAA AAG GCT TAT ATT AAT TTA

145 GGC TCC CGC TCA AAA GAA TTA ACT CCG AAC CGT TTT CGC AAA ATT GCT

193 GCA AAA TTA GGT CAT TAT CCC CGT GAT ATG CAA ATT AAT TTT GAT AAA

241 TTT CCA AAT TCT TTC TTG AGA TAT TTA ATC GAA GTT ATT GCT TTT CAA

289 AGA TCT GAT ATC TTC TCG CTA AGA GCC GAT TAT GCT AAA AAT CGA GCT

337 AAA AAT CGT GAT ATT TTA GTG GTT TCA AGT TAT TTA GAT GAA CTA AAA

385 CCG ATA ATT GAT AAA TAC CAA ATA ATT AAT AAC AGC GTC AAT TAT GCC

433 CGT TAT TAC CAA AAT ATG CCC CCT AAT ATG GCA AGT TCT GAA TTT CTT

481 GCA AGC GAG ATT CAG AAG AAA ATG AAC CTA AAT CCA AAA TTA ACG GTC

529 AAG GTT TTA GGC GAA AAT GAA GTG CGA AAA TTA GGA ATG AAC CTG CTT

577 TTA GCG GTC AAT AGA GGA TCA ACT TAT GAT GCT AAA TTA GTA GTG ATT

625 TCT TAT GAA GGG CTT CCA GGA TCA CAA TAT AAA ACT GCT TTT ATT GGT

673 AAA GGA ATT ACT TTT GAT TCA GGT GGT TAT AAT ATC AAA ACT GGC ATG

721 TAT ATG AAT GAT ATG AAA ATT GAT ATG TCA GGA GCG ATA ATC TGT GCA

769 GCT GCA ATT GAT GCC TTA TCA CAA TTT AAT CCC TTA GCA AAT GTT GTC

817 GCT GTT TTA CCA CTT ACC GAT AAC CGT CTT AAT GGC GAT GCA AAT ACG

865 CCC GAT GCA GTA TGA AGA TCA ATG AAT GGT AAA ACA GTT GAG ATT AAT

913 AAT ACT GAC GCC GAG GGT CGG CTC ATT TTA GCA GAT GCA ATC ACT TAT

961 GCG ATT AGA CAA GAA AAA GCA AGT GAA ATT ATT TCG ATT GCA ACT CTG

1009 ACA GGA GCA ATT CGT ATT GCT TTA GGG GAA ACC TTT ACT GGA GCT TTT

1057 GCA AAT GAG GAA AAA ATT TGA AAG AAC TTT AAC GAG GCA TCA AAA GAA

1105 GCC GGA GAA CTA ATT TGA AGA ATG CCT CTT CAT CAG GAT TTT GCC CAA

1153 AAT ATC CGT GAT TCA AAA GTG GCG GAT TTA AAA AAT ACT GAT TTT TCC

1201 GGC AAA GCC GGT TCT TCT TCT GCC GCA ATG TTT CTA GCT GAG TTT GTC

1249 GAA GAT AAA CCG TTT ATT CAT TTG GAT ATT GCC GCG ACT GCT TTT GTC

1297 AAA AAT ACC CCA ACT GGT GTT ATG GTT AGG TCT TTA GTT GAA TAT ATT

1345 TTA GCA AAG CAA AAC TAA
