# Supplementary material for: MHJ_0461 is a multifunctional leucine aminopeptidase on the surface of Mycoplasma hyopneumoniae
Source: Open Biol. 2015 Jan 14;5(1):140175. doi: 10.1098/rsob.140175 (PMC4313372; doi:10.1098/rsob.140175)
Supplement: Peptide coverage for rMHJ_0461 multimeric bands [file rsob140175supp2.pdf]

Mass Spectrometry data on rMHJ_0461 multimeric bands in denaturing gel

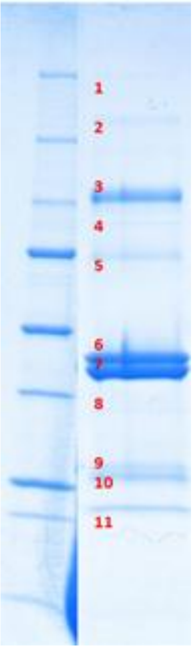

Figure above shows rMHJ_0461 multimeric bands, which are numbered 1 to 11. Mass spec data for each band is provided below.

Band 1 250kDa

2::Q4A9M4 **Mass:** 51376 **Score:** 707 **Matches:** 10(5) **Sequences:** 10(5) **emPAI:** 0.64 tr|Q4A9M4|pepA Leucyl aminopeptidase Tax_Id=262719 [Mycoplasma hyopneumoniae (strain J / ATCC 25934 / NCTC 10110)]

Protein sequence coverage: 30%

**1**	MKNFSEIFIK	YSNKFESNRI	TIEPAYSDSQ	IPMLIKEDLA	ITEYLGQKKA
**51**	YINLGSRSKE	LTPNRFRKIA	AKLGHYPRDM	QINFDKFPNS	FLRYLIEVIA
**101**	FQRSDIFSLR	ADYAKNRAKN	RDILVVSSYL	DELKPIIDKY	QIINNSVNYA
**151**	RYYQNMPPNM	ASSEFLASEI	QKKMNLNPKL	TVKVLGENEV	RKLGMNLLLA
**201**	VNRGSTYDAK	LVVISYEGLP	GSQYKTAFIG	KGITFDSGGY	NIKTGMYMND
**251**	MKIDMSGAII	CAAAIDALSQ	FNPLANVVAV	LPLTDNRLNG	DANTPDAVWR
**301**	SMNGKTVEIN	NTDAEGRLIL	ADAITYAIRQ	EKASEIISIA	TLTGAIRIAL
**351**	GETFTGAFAN	EEKIWKNFNE	ASKEAGELIW	RMPLHQDFAQ	NIRDSKVADL
**401**	KNTDFSGKAG	SSSAAMFLAE	FVEDKPFIHL	DIAATAFVKN	TPTGVMVRSL
**451**	VEYILAKQN				

Query	Start	–	End	Observed	Mr(expt)	Mr(calc)	ppm	M	Score	Expect	Rank	U	Peptide
261	20	–	36	968.0234	1934.0323	1934.0070	13.1	0	76	0.028	1	U	R.ITIEPAYSDSQIPMLIK.E + Oxidation (M)
164	37	–	48	690.3646	1378.7146	1378.6980	12.0	0	77	0.015	1	U	K.EDLAITEYLGQK.K
276	122	–	139	1030.5959	2059.1772	2059.1452	15.5	0	91	0.00081	1	U	R.DILVVSSYLDELKPIIDK.Y
186	140	–	151	727.8908	1453.7670	1453.7313	24.5	0	61	0.71	1	U	K.YQIINNSVNYAR.Y
123	277	–	287	598.8626	1195.7106	1195.6925	15.2	0	58	0.61	1	U	N.VVAVLPLTDNR.L
182	288	–	300	715.3578	1428.7010	1428.6633	26.4	0	52	5.4	1	U	R.LNGDANTPDAVWR.S + Deamidated (NQ)
153	318	–	329	666.9082	1331.8018	1331.7812	15.5	0	84	0.0018	1		R.LILADAITYAIR.Q
199	333	–	347	758.4526	1514.8906	1514.8668	15.7	0	106	1.5e-05	1	U	K.ASEIISIATLTGAIR.I

Query	Start	–	End	Observed	Mr(expt)	Mr(calc)	ppm	M	Score	Expect	Rank	U	Peptide
230	348	–	363	849.4329	1696.8512	1696.8308	12.0	0	60	0.99	1	U	R.IALGETFTGAFANEEK.I
194	382	–	393	743.3822	1484.7498	1484.7194	20.5	0	55	2.8	1	U	R.MPLHQDFAQNIR.D + Oxidation (M)

Band 2 175kDa

2::Q4A9M4 **Mass:** 51376 **Score:** 1423 **Matches:** 25(9) **Sequences:** 21(9) **emPAI:** 1.53 tr|Q4A9M4|pepA Leucyl aminopeptidase Tax_Id=262719 [Mycoplasma hyopneumoniae (strain J / ATCC 25934 / NCTC 10110)]

Protein sequence coverage: 52%

1	MK**NFSEIFIK**	YSNKFESNR**I**	**TIEPAYSDSQ**	**IPMLIKEDLA**	**ITEYLGQK**KA
51	YINLGSRSKE	LTPNRFRKIA	AKLGHYPR**DM**	**QINFDKFPNS**	**FLRYLIEVIA**
101	**FQR**SDIFSLR	ADYAKNRAKN	R**DILVVSSYL**	**DELKPIIDKY**	**QIINNSVNYA**
151	**RYYQNMPPNM**	**ASSEFLASEI**	**Q**KKMNLNPKL	TVKVLGENEV	R**KLGMNLLLA**
201	**VNR**GSTYDAK	**LVVISYEGLP**	**GSQYK**TAFIG	K**GITFDSGGY**	**NIK**TGMYMND
251	MKIDMSGAII	CAAAIDALSQ	FNPLAN**VVAV**	**LPLTDNRLNG**	**DANTPDAVWR**
301	SMNGKTVEIN	NTDAEGR**LIL**	**ADAITYAIR**Q	EK**ASEIISIA**	**TLTGAIRIAL**
351	**GETFTGAFAN**	**EEK**IWKNFNE	ASKEAGELIW	R**MPLHQDFAQ**	**NIR**DSKVADL
401	KNTDFSGKAG	SSSAAMFLAE	FVEDKPFIHL	DIAATAFVKN	TPTGVMVR**SL**
451	**VEYILAK**QN				

Query	Start	–	End	Observed	Mr(expt)	Mr(calc)	ppm	M	Score	Expect	Rank	U	Peptide
86	3	–	10	499.2757	996.5369	996.5280	8.89	0	46	13	4	U	K.NFSEIFIK.Y
279	20	–	36	960.0221	1918.0296	1918.0121	9.14	0	61	0.85	1	U	R.ITIEPAYSDSQIPMLIK.E
284	20	–	36	968.0264	1934.0382	1934.0070	16.1	0	52	6.2	1	U	R.ITIEPAYSDSQIPMLIK.E + Oxidation (M)
173	37	–	48	690.3646	1378.7146	1378.6980	12.0	0	82	0.0057	1	U	K.EDLAITEYLGQK.K
178	79	–	89	692.8195	1383.6244	1383.6129	8.30	1	61	0.51	1	U	R.DMQINFDKFPN.S + Oxidation (M)
277	79	–	93	944.4739	1886.9333	1886.8985	18.5	1	83	0.0054	1	U	R.DMQINFDKFPNSFLR.Y + Oxidation (M)
59	87	–	93	440.7499	879.4852	879.4603	28.3	0	44	22	2	U	K.FPNSFLR.Y
142	94	–	103	626.3664	1250.7182	1250.7023	12.7	0	69	0.066	1	U	R.YLIEVIAFQR.S
298	122	–	139	1030.5959	2059.1772	2059.1452	15.5	0	91	0.00071	1	U	R.DILVVSSYLDELKPIIDK.Y
198	140	–	151	727.8831	1453.7516	1453.7313	14.0	0	68	0.15	1	U	K.YQIINNSVNYAR.Y
199	140	–	151	728.3762	1454.7378	1454.7153	15.4	0	65	0.29	1	U	K.YQIINNSVNYAR.Y + Deamidated (NQ)
200	140	–	151	728.3787	1454.7429	1454.7153	18.9	0	61	0.68	1	U	K.YQIINNSVNYAR.Y + Deamidated (NQ)
325	152	–	172	1240.5819	2479.1492	2479.1035	18.4	0	81	0.0095	1	U	R.YYQNMPPNMASSEFLASEIQK.K + 2 Oxidation (M)
170	192	–	203	679.4134	1356.8122	1356.7911	15.5	1	88	0.00084	1	U	R.KLGMNLLLAVNR.G + Oxidation (M)
134	193	–	203	615.3640	1228.7135	1228.6962	14.1	0	96	0.00015	1	U	K.LGMNLLLAVNR.G + Oxidation (M)
242	211	–	225	826.9601	1651.9057	1651.8821	14.3	0	52	5.4	1	U	K.LVVISYEGLPGSQYK.T
148	232	–	243	636.3242	1270.6337	1270.6194	11.3	0	62	0.58	1		K.GITFDSGGYNIK.T
127	277	–	287	598.8672	1195.7199	1195.6925	22.9	0	51	2.5	1	U	N.VVAVLPLTDNR.L
192	288	–	300	715.3426	1428.6706	1428.6633	5.09	0	71	0.062	1	U	R.LNGDANTPDAVWR.S + Deamidated (NQ)
164	318	–	329	666.9033	1331.7921	1331.7812	8.11	0	89	0.00067	1		R.LILADAITYAIR.Q
211	333	–	347	758.4500	1514.8854	1514.8668	12.3	0	89	0.00079	1	U	K.ASEIISIATLTGAIR.I
246	348	–	363	849.4274	1696.8402	1696.8308	5.54	0	73	0.049	1	U	R.IALGETFTGAFANEEK.I
202	382	–	393	735.3812	1468.7478	1468.7245	15.8	0	49	11	1	U	R.MPLHQDFAQNIR.D
207	382	–	393	743.3796	1484.7447	1484.7194	17.0	0	48	14	1	U	R.MPLHQDFAQNIR.D + Oxidation (M)
93	449	–	457	518.3173	1034.6201	1034.6012	18.3	0	43	20	1	U	R.SLVEYILAK.Q

Band 3 125kDa

1. 2::Q4A9M4 **Mass:** 51376 **Score:** 2372 **Matches:** 63(30) **Sequences:** 35(15) **emPAI:** 3.99 tr|Q4A9M4|pepA Leucyl aminopeptidase Tax_Id=262719 [Mycoplasma hyopneumoniae (strain J / ATCC 25934 / NCTC 10110)]

Protein sequence coverage: 66%

1	MKNFSEIFIK	YSNKFESNRI	TIEPAYSDSQ	IPMLIKEDLA	ITEYLGQKKA
51	YINLGSRSKE	LTPNRFRKIA	AKLGHYPRDM	QINFDKFPNS	FLRYLIEVIA
101	FQRSDIFSLR	ADYAKNRAKN	RDILVVSSYL	DELKPIIDKY	QIINNSVNYA
151	RYYQNMPPNM	ASSEFLASEI	QKKMNLNPKL	TVKVLGENEV	RKLGMNLLLA
201	VNRGSTYDAK	LVVISYEGLP	GSQYKTAFIG	KGITFDSGGY	NIKTGMYMND
251	MKIDMSGAII	CAAAIDALSQ	FNPLANVVAV	LPLTDNRLNG	DANTPDAVWR
301	SMNGKTVEIN	NTDAEGRLIL	ADAITYAIRQ	EKASEIISIA	TLTGAIRIAL
351	GETFTGAFAN	EEKIWKNFNE	ASKEAGELIW	RMPLHQDFAQ	NIRDSKVADL
401	KNTDFSGKAG	SSSAAMFLAE	FVEDKPFIHL	DIAATAFVKN	TPTGVMVRSL
451	VEYILAKQN				

Query	Start	–	End	Observed	Mr(expt)	Mr(calc)	ppm	M	Score	Expect	Rank	U	Peptide
70	3	–	10	499.2778	996.5411	996.5280	13.1	0	36	1.3e+02	1	U	K.NFSEIFIK.Y
71	3	–	10	499.7687	997.5229	997.5120	10.9	0	62	0.35	2	U	K.NFSEIFIK.Y + Deamidated (NQ)
240	20	–	33	790.8911	1579.7676	1579.7440	15.0	0	41	66	1	U	R.ITIEPAYSDSQIPM.L + Oxidation (M)
300	20	–	36	968.0205	1934.0264	1934.0070	10.0	0	32	6.3e+02	1	U	R.ITIEPAYSDSQIPMLIK.E + Oxidation (M)
270	34	–	48	867.4964	1732.9782	1732.9611	9.91	1	78	0.014	1	U	M.LIKEDLAITEYLGQK.K
148	37	–	48	690.3596	1378.7046	1378.6980	4.82	0	71	0.075	1	U	K.EDLAITEYLGQK.K
149	79	–	89	692.8220	1383.6294	1383.6129	11.9	1	57	1.4	1	U	R.DMQINFDKFPN.S + Oxidation (M)
249	79	–	91	809.8728	1617.7311	1617.7133	10.9	1	58	1.3	1	U	R.DMQINFDKFPNSF.L + Oxidation (M)
294	79	–	93	944.9657	1887.9169	1887.8825	18.2	1	60	1	1	U	R.DMQINFDKFPNSFLR.Y + Deamidated (NQ); Oxidation (M)
116	94	–	103	626.3664	1250.7182	1250.7023	12.7	0	69	0.073	1	U	R.YLIEVIAFQR.S
69	103	–	110	497.3145	992.6145	992.5403	74.7	1	35	76	2	U	Q.RSDIFSLR.A
28	104	–	110	419.2311	836.4476	836.4392	10.0	0	49	6.7	1	U	R.SDIFSLR.A
314	122	–	139	1030.5777	2059.1408	2059.1452	-2.17	0	83	0.0057	1	U	R.DILVVSSYLDELKPIIDK.Y
315	122	–	139	1030.5898	2059.1651	2059.1452	9.64	0	94	0.00035	1	U	R.DILVVSSYLDELKPIIDK.Y
316	122	–	139	1030.5929	2059.1712	2059.1452	12.6	0	82	0.0053	1	U	R.DILVVSSYLDELKPIIDK.Y
317	122	–	139	1030.5929	2059.1712	2059.1452	12.6	0	65	0.32	1	U	R.DILVVSSYLDELKPIIDK.Y
178	140	–	151	728.3710	1454.7275	1454.7153	8.38	0	63	0.42	1	U	K.YQIINNSVNYAR.Y + Deamidated (NQ)
179	140	–	151	728.3736	1454.7326	1454.7153	11.9	0	56	2.3	1	U	K.YQIINNSVNYAR.Y + Deamidated (NQ)
348	152	–	172	1240.5719	2479.1292	2479.1035	10.4	0	42	78	1	U	R.YYQNMPPNMASSEFLASEIQK.K + 2 Oxidation (M)
297	156	–	172	956.4584	1910.9022	1910.8754	14.0	0	85	0.0033	1	U	N.MPPNMASSEFLASEIQK.K + 2 Oxidation (M)
143	192	–	203	679.4084	1356.8023	1356.7911	8.27	1	88	0.00098	1	U	R.KLGMNLLLAVNR.G + Oxidation (M)
112	193	–	203	615.3593	1228.7041	1228.6962	6.50	0	83	0.0033	1	U	K.LGMNLLLAVNR.G + Oxidation (M)
253	211	–	225	826.9574	1651.9003	1651.8821	11.0	0	62	0.52	1	U	K.LVVISYEGLPGSQYK.T
121	232	–	243	636.3242	1270.6337	1270.6194	11.3	0	65	0.26	1		K.GITFDSGGYNIK.T
355	264	–	287	1276.2211	2550.4277	2550.3806	18.5	0	70	0.12	1	U	A.AIDALSQFNPLANVVAVLPLTDNR.L
356	264	–	287	1276.2211	2550.4277	2550.3806	18.5	0	95	0.00037	1	U	A.AIDALSQFNPLANVVAVLPLTDNR.L
242	273	–	287	796.4628	1590.9111	1590.9093	1.11	0	88	0.0011	1	U	N.PLANVVAVLPLTDNR.L
104	277	–	287	598.8580	1195.7014	1195.6925	7.43	0	73	0.02	1	U	N.VVAVLPLTDNR.L
164	288	–	300	714.8641	1427.7136	1427.6793	24.0	0	53	4.2	1	U	R.LNGDANTPDAVWR.S
165	288	–	300	715.3426	1428.6706	1428.6633	5.09	0	86	0.0021	1	U	R.LNGDANTPDAVWR.S + Deamidated (NQ)

Query	Start	–	End	Observed	Mr(expt)	Mr(calc)	ppm	M	Score	Expect	Rank	U	Peptide
166	288	–	300	715.3502	1428.6858	1428.6633	15.7	0	55	2.7	1	U	R.LNGDANTPDAVWR.S + Deamidated (NQ)
167	288	–	300	715.3527	1428.6909	1428.6633	19.3	0	101	7.8e-05	1	U	R.LNGDANTPDAVWR.S + Deamidated (NQ)
136	318	–	329	666.9033	1331.7921	1331.7812	8.11	0	50	5.5	1		R.LILADAITYAIR.Q
137	318	–	329	666.9033	1331.7921	1331.7812	8.11	0	70	0.054	1		R.LILADAITYAIR.Q
138	318	–	329	666.9057	1331.7969	1331.7812	11.8	0	83	0.0029	1		R.LILADAITYAIR.Q
139	318	–	329	666.9082	1331.8018	1331.7812	15.5	0	91	0.0004	1		R.LILADAITYAIR.Q
267	318	–	332	859.9922	1717.9698	1717.9614	4.91	1	35	2.5e+02	3	U	R.LILADAITYAIRQEK.A + Deamidated (NQ)
296	330	–	347	951.5399	1901.0652	1901.0469	9.64	1	72	0.055	1	U	R.QEKASEIISIATLTGAIR.I + Deamidated (NQ)
207	333	–	347	758.4447	1514.8749	1514.8668	5.39	0	85	0.0025	1	U	K.ASEIISIATLTGAIR.I
208	333	–	347	758.4447	1514.8749	1514.8668	5.39	0	130	8e-08	1	U	K.ASEIISIATLTGAIR.I
209	333	–	347	758.4474	1514.8802	1514.8668	8.84	0	110	6.4e-06	1	U	K.ASEIISIATLTGAIR.I
210	333	–	347	758.4474	1514.8802	1514.8668	8.84	0	92	0.00045	1	U	K.ASEIISIATLTGAIR.I
211	333	–	347	758.4500	1514.8854	1514.8668	12.3	0	72	0.041	1	U	K.ASEIISIATLTGAIR.I
212	333	–	347	758.4500	1514.8854	1514.8668	12.3	0	66	0.18	1	U	K.ASEIISIATLTGAIR.I
213	333	–	347	758.4526	1514.8906	1514.8668	15.7	0	92	0.00039	1	U	K.ASEIISIATLTGAIR.I
214	333	–	347	758.4526	1514.8906	1514.8668	15.7	0	113	3.1e-06	1	U	K.ASEIISIATLTGAIR.I
215	333	–	347	758.4552	1514.8958	1514.8668	19.2	0	93	0.0003	1	U	K.ASEIISIATLTGAIR.I
216	333	–	347	758.4578	1514.9010	1514.8668	22.6	0	85	0.0019	1	U	K.ASEIISIATLTGAIR.I
257	348	–	363	849.3887	1696.7629	1696.8308	-40.0	0	62	0.57	1	U	R.IALGETFTGAFANEEK.I
258	348	–	363	849.4191	1696.8236	1696.8308	-4.22	0	80	0.0094	1	U	R.IALGETFTGAFANEEK.I
259	348	–	363	849.4274	1696.8402	1696.8308	5.54	0	64	0.37	1	U	R.IALGETFTGAFANEEK.I
260	348	–	363	849.4274	1696.8402	1696.8308	5.54	0	78	0.014	1	U	R.IALGETFTGAFANEEK.I
261	348	–	363	849.4356	1696.8567	1696.8308	15.3	0	48	17	1	U	R.IALGETFTGAFANEEK.I
262	348	–	363	849.4384	1696.8623	1696.8308	18.5	0	83	0.0046	1	U	R.IALGETFTGAFANEEK.I
61	374	–	381	487.2716	972.5287	972.5029	26.6	0	55	2.6	1		K.EAGELIWR.M
190	382	–	393	743.3744	1484.7343	1484.7194	10.0	0	46	22	1	U	R.MPLHQDFAQNIR.D + Oxidation (M)
192	382	–	393	743.8701	1485.7257	1485.7034	15.0	0	54	4.1	1	U	R.MPLHQDFAQNIR.D + Deamidated (NQ); Oxidation (M)
194	382	–	393	743.8805	1485.7464	1485.7034	28.9	0	25	3.1e+03	8	U	R.MPLHQDFAQNIR.D + Deamidated (NQ); Oxidation (M)
246	382	–	394	800.8882	1599.7619	1599.7464	9.71	1	54	3.7	1	U	R.MPLHQDFAQNIRD.S + Oxidation (M)
277	409	–	425	888.4287	1774.8429	1774.8084	19.5	0	121	7.6e-07	1	U	K.AGSSSAAMFLAEFVEDK.P + Oxidation (M)
311	409	–	427	1010.4895	2018.9645	2018.9295	17.3	0	83	0.0055	1	U	K.AGSSSAAMFLAEFVEDKPF.I + Oxidation (M)
347	418	–	439	1237.6691	2473.3237	2473.3257	-0.79	0	51	11	1	U	F.LAEFVEDKPFIHLDIAATAFVK.N
78	449	–	457	518.3130	1034.6115	1034.6012	10.0	0	43	22	1	U	R.SLVEYILAK.Q

Band 4 87kDa

2::Q4A9M4 **Mass:** 51376 **Score:** 1473 **Matches:** 25(9) **Sequences:** 21(8) **emPAI:** 1.23 tr|Q4A9M4|pepA Leucyl aminopeptidase Tax_Id=262719 [Mycoplasma hyopneumoniae (strain J / ATCC 25934 / NCTC 10110)]

Protein sequence coverage: 53%

1	MKNFSEIFIK	YSNKFESNRI	TIEPAYSDSQ	IPMLIKEDLA	ITEYLGQKKA
51	YINLGSRSKE	LTPNRFRKIA	AKLGHYPRDM	QINFDKFPNS	FLRYLIEVIA
101	FQRSDIFSLR	ADYAKNRAKN	RDILVVSSYL	DELKPIIDKY	QIINNSVNYA
151	RYYQNMPPNM	ASSEFLASEI	QKKMNLNPKL	TVKVLGENEV	RKLGMNLLLA
201	VNRGSTYDAK	LVVISYEGLP	GSQYKTAFIG	KGITFDSGGY	NIKTGMYMND
251	MKIDMSGAII	CAAAIDALSQ	FNPLANVVAV	LPLTDNRLNG	DANTPDAVWR
301	SMNGKTVEIN	NTDAEGRLIL	ADAITYAIRQ	EKASEIISIA	TLTGAIRIAL

**351**	GETFTGAFAN	EEKIWKNFNE	ASKEAGELIW	RMPLHQDFAQ	NIRDSKVADL
**401**	KNTDFSGKAG	SSSAAMFLAE	FVEDKPFIHL	DIAATAFVKN	TPTGVMVRSL
**451**	VEYILAKQN				

Query	Start	–	End	Observed	Mr(expt)	Mr(calc)	ppm	M	Score	Expect	Rank	U	Peptide
90	3	–	10	499.2757	996.5369	996.5280	8.89	0	46	13	1	U	K.NFSEIFIK.Y
293	20	–	36	968.0205	1934.0264	1934.0070	10.0	0	53	6.1	1	U	R.ITIEPAYSDSQIPMLIK.E + Oxidation (M)
185	37	–	48	690.3646	1378.7146	1378.6980	12.0	0	48	14	3	U	K.EDLAITEYLGQK.K
189	79	–	89	692.8269	1383.6393	1383.6129	19.1	1	63	0.38	1	U	R.DMQINFDKFPN.S + Oxidation (M)
60	87	–	93	440.7479	879.4812	879.4603	23.8	0	39	63	6	U	K.FPNSFLR.Y
148	94	–	103	626.3688	1250.7230	1250.7023	16.5	0	51	4.3	1	U	R.YLIEVIAFQR.S
37	104	–	110	419.2311	836.4476	836.4392	10.0	0	56	1.4	1	U	R.SDIFSLR.A
301	122	–	139	1030.5959	2059.1772	2059.1452	15.5	0	91	0.00069	1	U	R.DILVVSSYLDELKPIIDK.Y
211	140	–	151	727.8805	1453.7465	1453.7313	10.5	0	77	0.018	1	U	K.YQIINNSVNYAR.Y
212	140	–	151	728.3685	1454.7224	1454.7153	4.87	0	45	31	2	U	K.YQIINNSVNYAR.Y + Deamidated (NQ)
334	152	–	172	1240.5719	2479.1292	2479.1035	10.4	0	69	0.15	1	U	R.YYQNMPPNMASSEFLASEIQK.K + 2 Oxidation (M)
180	192	–	203	679.4084	1356.8023	1356.7911	8.27	1	112	3.4e-06	1	U	R.KLGMNLLLAVNR.G + Oxidation (M)
143	193	–	203	607.3676	1212.7206	1212.7012	16.0	0	95	0.00016	1	U	K.LGMNLLLAVNR.G
144	193	–	203	615.3640	1228.7135	1228.6962	14.1	0	91	0.00053	1	U	K.LGMNLLLAVNR.G + Oxidation (M)
258	211	–	225	826.9574	1651.9003	1651.8821	11.0	0	65	0.31	1	U	K.LVVISYEGLPGSQYK.T
155	232	–	243	636.3242	1270.6337	1270.6194	11.3	0	65	0.26	1		K.GITFDSGGYNIK.T
139	277	–	287	598.8603	1195.7060	1195.6925	11.3	0	54	1.5	1	U	N.VVAVLPLTDNR.L
203	288	–	300	714.8565	1427.6984	1427.6793	13.4	0	28	1.3e+03	6	U	R.LNGDANTPDAVWR.S
204	288	–	300	715.3477	1428.6807	1428.6633	12.2	0	113	4.8e-06	1	U	R.LNGDANTPDAVWR.S + Deamidated (NQ)
173	318	–	329	666.9082	1331.8018	1331.7812	15.5	0	103	2.4e-05	1		R.LILADAITYAIR.Q
225	333	–	347	758.4474	1514.8802	1514.8668	8.84	0	119	8.6e-07	1	U	K.ASEIISIATLTGAIR.I
261	348	–	363	849.4356	1696.8567	1696.8308	15.3	0	90	0.001	1	U	R.IALGETFTGAFANEEK.I
215	382	–	393	735.3837	1468.7529	1468.7245	19.3	0	48	15	1	U	R.MPLHQDFAQNIR.D
220	382	–	393	743.3796	1484.7447	1484.7194	17.0	0	49	13	1	U	R.MPLHQDFAQNIR.D + Oxidation (M)
98	449	–	457	518.3130	1034.6115	1034.6012	10.0	0	44	15	1	U	R.SLVEYILAK.Q

Band 5 75kDa

2::Q4A9M4 **Mass:** 51376 **Score:** 1644 **Matches:** 37(13) **Sequences:** 25(10) **emPAI:** 2.45 tr|Q4A9M4|pepA Leucyl aminopeptidase Tax_Id=262719 [Mycoplasma hyopneumoniae (strain J / ATCC 25934 / NCTC 10110)]

Protein sequence coverage: 57%

**1**	MKNFSEIFIK	YSNKFESNRI	TIEPAYSDSQ	IPMLIKEDLA	ITEYLGQKKA
**51**	YINLGSRSKE	LTPNRFRKIA	AKLGHYPRDM	QINFDKFPNS	FLRYLIEVIA
**101**	FQRSDIFSLR	ADYAKNRAKN	RDILVVSSYL	DELKPIIDKY	QIINNSVNYA
**151**	RYYQNMPPNM	ASSEFLASEI	QKKMNLNPKL	TVKVLGENEV	RKLGMNLLLA
**201**	VNRGSTYDAK	LVVISYEGLP	GSQYKTAFIG	KGITFDSGGY	NIKTGMYMND
**251**	MKIDMSGAII	CAAAIDALSQ	FNPLANVVAV	LPLTDNRLNG	DANTPDAVWR
**301**	SMNGKTVEIN	NTDAEGRLIL	ADAITYAIRQ	EKASEIISIA	TLTGAIRIAL
**351**	GETFTGAFAN	EEKIWKNFNE	ASKEAGELIW	RMPLHQDFAQ	NIRDSKVADL
**401**	KNTDFSGKAG	SSSAAMFLAE	FVEDKPFIHL	DIAATAFVKN	TPTGVMVRSL
**451**	VEYILAKQN				

Query	Start	–	End	Observed	Mr(expt)	Mr(calc)	ppm	M	Score	Expect	Rank	U	Peptide
100	3	–	10	499.2778	996.5411	996.5280	13.1	0	41	38	1	U	K.NFSEIFIK.Y
304	20	–	36	960.0309	1918.0472	1918.0121	18.3	0	65	0.33	1	U	R.ITIEPAYSDSQIPMLIK.E
307	20	–	36	968.0264	1934.0382	1934.0070	16.1	0	48	16	1	U	R.ITIEPAYSDSQIPMLIK.E + Oxidation (M)
183	37	–	48	690.3646	1378.7146	1378.6980	12.0	0	75	0.024	1	U	K.EDLAITEYLGQK.K
78	50	–	57	447.2532	892.4919	892.4766	17.1	0	36	1.4e+02	1	U	K.AYINLGSR.S
186	79	–	89	692.8319	1383.6493	1383.6129	26.3	1	52	4.7	1	U	R.DMQINFDKFPN.S + Oxidation (M)
301	79	–	93	944.4710	1886.9275	1886.8985	15.4	1	76	0.025	1	U	R.DMQINFDKFPNSFLR.Y + Oxidation (M)
73	87	–	93	440.7439	879.4732	879.4603	14.7	0	40	56	3	U	K.FPNSFLR.Y
153	94	–	103	626.3664	1250.7182	1250.7023	12.7	0	52	3.2	4	U	R.YLIEVIAFQR.S
45	104	–	110	419.2350	836.4554	836.4392	19.3	0	51	3.3	1	U	R.SDIFSLR.A
318	122	–	139	1030.5959	2059.1772	2059.1452	15.5	0	85	0.0029	1	U	R.DILVVSSYLDELKPIIDK.Y
205	140	–	151	727.8805	1453.7465	1453.7313	10.5	0	61	0.7	1	U	K.YQIINNSVNYAR.Y
206	140	–	151	727.8857	1453.7568	1453.7313	17.5	0	90	0.00082	1	U	K.YQIINNSVNYAR.Y
207	140	–	151	728.3762	1454.7378	1454.7153	15.4	0	65	0.31	1	U	K.YQIINNSVNYAR.Y + Deamidated (NQ)
208	140	–	151	728.3813	1454.7480	1454.7153	22.4	0	44	36	1	U	K.YQIINNSVNYAR.Y + Deamidated (NQ)
209	140	–	151	728.3864	1454.7582	1454.7153	29.5	0	56	2.2	1	U	K.YQIINNSVNYAR.Y + Deamidated (NQ)
342	152	–	172	1240.5819	2479.1492	2479.1035	18.4	0	58	2.1	1	U	R.YYQNMPPNMASSEFLASEIQK.K + 2 Oxidation (M)
176	192	–	203	671.4120	1340.8094	1340.7962	9.87	1	92	0.0003	1	U	R.KLGMNLLLAVNR.G
179	192	–	203	679.4158	1356.8171	1356.7911	19.2	1	98	7.8e-05	1	U	R.KLGMNLLLAVNR.G + Oxidation (M)
150	193	–	203	607.3676	1212.7206	1212.7012	16.0	0	112	3.2e-06	1	U	K.LGMNLLLAVNR.G
151	193	–	203	615.3664	1228.7182	1228.6962	18.0	0	64	0.26	1	U	K.LGMNLLLAVNR.G + Oxidation (M)
264	211	–	225	826.9574	1651.9003	1651.8821	11.0	0	68	0.14	1	U	K.LVVISYEGLPGSQYK.T
157	232	–	243	636.3289	1270.6433	1270.6194	18.8	0	49	11	1		K.GITFDSGGYNIK.T
144	277	–	287	598.8649	1195.7153	1195.6925	19.1	0	64	0.13	1	U	N.VVAVLPLTDNR.L
198	288	–	300	714.8590	1427.7035	1427.6793	16.9	0	86	0.002	1	U	R.LNGDANTPDAVWR.S
199	288	–	300	714.8616	1427.7086	1427.6793	20.5	0	53	5	1	U	R.LNGDANTPDAVWR.S
200	288	–	300	715.3451	1428.6757	1428.6633	8.64	0	95	0.00028	1	U	R.LNGDANTPDAVWR.S + Deamidated (NQ)
201	288	–	300	715.3477	1428.6807	1428.6633	12.2	0	98	0.00016	1	U	R.LNGDANTPDAVWR.S + Deamidated (NQ)
174	318	–	329	666.9131	1331.8116	1331.7812	22.8	0	89	0.00054	1		R.LILADAITYAIR.Q
302	330	–	347	951.5486	1901.0827	1901.0469	18.9	1	70	0.096	1	U	R.QEKASEIISIATLTGAIR.I + Deamidated (NQ)
226	333	–	347	758.4526	1514.8906	1514.8668	15.7	0	103	3.5e-05	1	U	K.ASEIISIATLTGAIR.I
270	348	–	363	849.9214	1697.8282	1697.8148	7.86	0	32	6.5e+02	2	U	R.IALGETFTGAFANEEK.I + Deamidated (NQ)
96	374	–	381	487.2696	972.5246	972.5029	22.3	0	49	9.7	1		K.EAGELIWR.M
213	382	–	393	735.3812	1468.7478	1468.7245	15.8	0	76	0.022	1	U	R.MPLHQDFAQNIR.D
218	382	–	393	743.3770	1484.7395	1484.7194	13.5	0	52	5.5	1	U	R.MPLHQDFAQNIR.D + Oxidation (M)
219	382	–	393	743.8675	1485.7205	1485.7034	11.5	0	35	3.2e+02	1	U	R.MPLHQDFAQNIR.D + Deamidated (NQ); Oxidation (M)
107	449	–	457	518.3173	1034.6201	1034.6012	18.3	0	48	6.6	1	U	R.SLVEYILAK.Q

Band 6 47 kDa

2::Q4A9M4 **Mass:** 51376 **Score:** 3765 **Matches:** 82(39) **Sequences:** 51(25) **emPAI:** 14.21 tr|Q4A9M4|pepA Leucyl aminopeptidase Tax_Id=262719 [Mycoplasma hyopneumoniae (strain J / ATCC 25934 / NCTC 10110)]

Protein sequence coverage: 68%

1 MK**NFSEIFIK** YSNKFESNRI **TIEPAYSDSQ** **IPMLIKEDLA** **ITEYLGQK**KA

51	YINLGSRSKE	LTPNRFRKIA	AKLGHYPRDM	QINFDKFPNS	FLRYLIEVIA
101	FQRSDIFSLR	ADYAKNRAKN	RDILVVSSYL	DELKPIIDKY	QIINNSVNYA
151	RYYQNMPPNM	ASSEFLASEI	QKKMNLNPKL	TVKVLGENEV	RKLGMNLLLA
201	VNRGSTYDAK	LVVISYEGLP	GSQYKTAFIG	KGITFDSGGY	NIKTGMYMND
251	MKIDMSGAII	CAAAIDALSQ	FNPLANVVAV	LPLTDNRLNG	DANTPDAVWR
301	SMNGKTVEIN	NTDAEGRLIL	ADAITYAIRQ	EKASEIISIA	TLTGAIRIAL
351	GETFTGAFAN	EEKIWKNFNE	ASKEAGELIW	RMPLHQDFAQ	NIRDSKVADL
401	KNTDFSGKAG	SSSAAMFLAE	FVEDKPFIHL	DIAATAFVKN	TPTGVMVRSL
451	VEYILAKQN				

Query	Start	–	End	Observed	Mr(expt)	Mr(calc)	ppm	M	Score	Expect	Rank	U	Peptide
79	3	–	10	499.2778	996.5411	996.5280	13.1	0	44	18	1	U	K.NFSEIFIK.Y
269	20	–	33	782.8919	1563.7693	1563.7490	13.0	0	36	2.6e+02	1	U	R.ITIEPAYSDSQIPM.L
277	20	–	33	790.8884	1579.7623	1579.7440	11.6	0	51	8	1	U	R.ITIEPAYSDSQIPM.L + Oxidation (M)
335	20	–	36	960.0162	1918.0179	1918.0121	3.02	0	70	0.11	1	U	R.ITIEPAYSDSQIPMLIK.E
338	20	–	36	968.0264	1934.0382	1934.0070	16.1	0	61	0.85	1	U	R.ITIEPAYSDSQIPMLIK.E + Oxidation (M)
146	26	–	36	655.8374	1309.6603	1309.6588	1.21	0	69	0.099	1	U	A.YSDSQIPMLIK.E + Oxidation (M)
315	34	–	48	867.4936	1732.9727	1732.9611	6.69	1	68	0.13	1	U	M.LIKEDLAITEYLGQK.K
168	37	–	48	690.3671	1378.7196	1378.6980	15.6	0	81	0.0061	1	U	K.EDLAITEYLGQK.K
54	50	–	57	447.2532	892.4919	892.4766	17.1	0	29	6.6e+02	1	U	K.AYINLGSR.S
85	79	–	86	505.7402	1009.4658	1009.4539	11.8	0	48	7.9	1	U	R.DMQINFDK.F
169	79	–	89	692.8220	1383.6294	1383.6129	11.9	1	61	0.56	1	U	R.DMQINFDKFPN.S + Oxidation (M)
288	79	–	91	801.8799	1601.7452	1601.7184	16.7	1	44	42	1	U	R.DMQINFDKFPNSF.L
293	79	–	91	809.8701	1617.7257	1617.7133	7.61	1	56	2.2	1	U	R.DMQINFDKFPNSF.L + Oxidation (M)
327	79	–	93	936.9619	1871.9093	1871.8876	11.6	1	71	0.094	1	U	R.DMQINFDKFPNSFLR.Y + Deamidated (NQ)
329	79	–	93	944.9628	1887.9111	1887.8825	15.1	1	47	23	1	U	R.DMQINFDKFPNSFLR.Y + Deamidated (NQ); Oxidation (M)
128	94	–	103	626.3617	1250.7088	1250.7023	5.17	0	66	0.13	1	U	R.YLIEVIAFQR.S
222	122	–	134	747.4171	1492.8196	1492.8025	11.5	0	93	0.00041	1	U	R.DILVVSSYLDELK.P
351	122	–	139	1030.5807	2059.1469	2059.1452	0.78	0	46	28	2	U	R.DILVVSSYLDELKPIIDK.Y
352	122	–	139	1030.5837	2059.1529	2059.1452	3.73	0	79	0.012	1	U	R.DILVVSSYLDELKPIIDK.Y
353	122	–	139	1030.5898	2059.1651	2059.1452	9.64	0	100	8.7e-05	1	U	R.DILVVSSYLDELKPIIDK.Y
366	122	–	140	1112.1210	2222.2275	2222.2086	8.52	1	51	7.9	1	U	R.DILVVSSYLDELKPIIDKY.Q
156	129	–	139	673.8944	1345.7743	1345.7493	18.6	0	63	0.37	1	U	S.YLDELKPIIDK.Y
117	130	–	139	592.3607	1182.7069	1182.6860	17.7	0	54	1.6	4	U	Y.LDELKPIIDK.Y
121	140	–	149	614.3100	1226.6055	1226.5931	10.0	0	27	1.6e+03	7	U	K.YQIINNSVNY.A
198	140	–	151	727.8805	1453.7465	1453.7313	10.5	0	50	9.7	1	U	K.YQIINNSVNYAR.Y
199	140	–	151	728.3710	1454.7275	1454.7153	8.38	0	90	0.00093	1	U	K.YQIINNSVNYAR.Y + Deamidated (NQ)
392	152	–	172	1224.5843	2447.1541	2447.1137	16.5	0	45	46	1	U	R.YYQNMPPNMASSEFLASEIQK.K
395	152	–	172	1232.5751	2463.1356	2463.1086	11.0	0	59	1.5	1	U	R.YYQNMPPNMASSEFLASEIQK.K + Oxidation (M)
398	152	–	172	1240.5752	2479.1359	2479.1035	13.0	0	59	1.7	1	U	R.YYQNMPPNMASSEFLASEIQK.K + 2 Oxidation (M)
328	156	–	172	940.4543	1878.8941	1878.8855	4.55	0	74	0.042	1	U	N.MPPNMASSEFLASEIQK.K
330	156	–	172	948.4584	1894.9023	1894.8804	11.5	0	86	0.0027	1	U	N.MPPNMASSEFLASEIQK.K + Oxidation (M)
331	156	–	172	948.4613	1894.9081	1894.8804	14.6	0	79	0.013	1	U	N.MPPNMASSEFLASEIQK.K + Oxidation (M)
333	156	–	172	956.4613	1910.9081	1910.8754	17.1	0	76	0.026	1	U	N.MPPNMASSEFLASEIQK.K + 2 Oxidation (M)
191	160	–	172	720.8606	1439.7067	1439.6966	7.00	0	111	7e-06	1	U	N.MASSEFLASEIQK.K
144	161	–	172	655.3357	1308.6568	1308.6561	0.51	0	37	1.7e+02	3	U	M.ASSEFLASEIQK.K
155	192	–	203	671.4071	1340.7996	1340.7962	2.55	1	109	6.1e-06	1	U	R.KLGMNLLLAVNR.G
159	192	–	203	679.4084	1356.8023	1356.7911	8.27	1	102	3.9e-05	1	U	R.KLGMNLLLAVNR.G + Oxidation (M)
120	193	–	203	607.3606	1212.7066	1212.7012	4.42	0	88	0.00096	1	U	K.LGMNLLLAVNR.G
123	193	–	203	615.3593	1228.7041	1228.6962	6.50	0	77	0.014	1	U	K.LGMNLLLAVNR.G + Oxidation (M)
56	196	–	203	456.7920	911.5695	911.5552	15.7	0	52	1.5	1	U	M.NLLLAVNR.G

Query	Start	–	End	Observed	Mr(expt)	Mr(calc)	ppm	M	Score	Expect	Rank	U	Peptide
296	211	–	225	826.9574	1651.9003	1651.8821	11.0	0	71	0.074	1	U	K.LVVISYEGLPGSQYK.T
297	211	–	225	826.9601	1651.9057	1651.8821	14.3	0	58	1.4	1	U	K.LVVISYEGLPGSQYK.T
132	232	–	243	636.3361	1270.6576	1270.6194	30.1	0	61	0.7	1		K.GITFDSGGYNIK.T
415	262	–	287	1347.2512	2692.4879	2692.4548	12.3	0	89	0.0017	1	U	C.AAAIDALSQFNPLANVVAVLPLTDNR.L
410	263	–	287	1311.7272	2621.4398	2621.4177	8.44	0	103	5.7e-05	1	U	A.AAIDALSQFNPLANVVAVLPLTDNR.L
406	264	–	287	1276.2143	2550.4141	2550.3806	13.2	0	129	1.6e-07	1	U	A.AIDALSQFNPLANVVAVLPLTDNR.L
372	267	–	287	1126.6441	2251.2737	2251.2325	18.3	0	109	1.4e-05	1	U	D.ALSQFNPLANVVAVLPLTDNR.L
354	269	–	287	1034.5660	2067.1175	2067.1113	3.01	0	111	8.8e-06	1	U	L.SQFNPLANVVAVLPLTDNR.L
284	273	–	287	796.4682	1590.9218	1590.9093	7.83	0	97	0.00016	1	U	N.PLANVVAVLPLTDNR.L
118	277	–	287	598.8603	1195.7060	1195.6925	11.3	0	72	0.025	1		N.VVAVLPLTDNR.L
184	288	–	300	714.8565	1427.6984	1427.6793	13.4	0	79	0.012	1	U	R.LNGDANTPDAVWR.S
185	288	–	300	715.3426	1428.6706	1428.6633	5.09	0	68	0.14	1	U	R.LNGDANTPDAVWR.S + Deamidated (NQ)
186	288	–	300	715.3451	1428.6757	1428.6633	8.64	0	77	0.018	1	U	R.LNGDANTPDAVWR.S + Deamidated (NQ)
150	318	–	329	666.8935	1331.7725	1331.7812	-6.58	0	78	0.012	1		R.LILADAITYAIR.Q
151	318	–	329	666.9033	1331.7921	1331.7812	8.11	0	41	40	3		R.LILADAITYAIR.Q
152	318	–	329	666.9057	1331.7969	1331.7812	11.8	0	60	0.57	1		R.LILADAITYAIR.Q
312	318	–	332	859.9950	1717.9754	1717.9614	8.14	1	31	6e+02	10	U	R.LILADAITYAIRQEK.A + Deamidated (NQ)
332	330	–	347	951.5311	1901.0477	1901.0469	0.42	1	83	0.0046	1	U	R.QEKASEIISIATLTGAIR.I + Deamidated (NQ)
234	333	–	347	758.4004	1514.7863	1514.8668	-53.1	0	116	2.2e-06	1	U	K.ASEIISIATLTGAIR.I
235	333	–	347	758.4421	1514.8697	1514.8668	1.95	0	64	0.27	1	U	K.ASEIISIATLTGAIR.I
236	333	–	347	758.4474	1514.8802	1514.8668	8.84	0	101	5.2e-05	1	U	K.ASEIISIATLTGAIR.I
237	333	–	347	758.4500	1514.8854	1514.8668	12.3	0	86	0.0019	1	U	K.ASEIISIATLTGAIR.I
238	333	–	347	758.4526	1514.8906	1514.8668	15.7	0	86	0.0015	1	U	K.ASEIISIATLTGAIR.I
239	333	–	347	758.4552	1514.8958	1514.8668	19.2	0	120	6.4e-07	1	U	K.ASEIISIATLTGAIR.I
122	336	–	347	614.8921	1227.7696	1227.7551	11.9	0	89	0.00045	1	U	E.IISIATLTGAIR.I
306	348	–	363	849.4219	1696.8292	1696.8308	-0.97	0	98	0.00017	1	U	R.IALGETFTGAFANEEK.I
307	348	–	363	849.4246	1696.8347	1696.8308	2.28	0	66	0.23	1	U	R.IALGETFTGAFANEEK.I
308	348	–	363	849.4412	1696.8678	1696.8308	21.8	0	74	0.037	1	U	R.IALGETFTGAFANEEK.I
282	349	–	363	792.8919	1583.7692	1583.7467	14.2	0	118	1.6e-06	1	U	I.ALGETFTGAFANEEK.I
68	374	–	381	487.2675	972.5204	972.5029	18.0	0	58	1.2	1		K.EAGELIWR.M
218	382	–	393	743.3796	1484.7447	1484.7194	17.0	0	38	1.5e+02	1	U	R.MPLHQDFAQNIR.D + Oxidation (M)
219	382	–	393	743.8650	1485.7154	1485.7034	8.04	0	25	2.8e+03	1	U	R.MPLHQDFAQNIR.D + Deamidated (NQ); Oxidation (M)
220	382	–	393	743.8701	1485.7257	1485.7034	15.0	0	61	0.81	1	U	R.MPLHQDFAQNIR.D + Deamidated (NQ); Oxidation (M)
281	382	–	394	792.8892	1583.7639	1583.7514	7.84	1	50	8.7	1	U	R.MPLHQDFAQNIRD.S
287	382	–	394	800.8989	1599.7833	1599.7464	23.1	1	54	3.9	1	U	R.MPLHQDFAQNIRD.S + Oxidation (M)
316	409	–	425	880.4185	1758.8225	1758.8134	5.16	0	95	0.00037	1	U	K.AGSSSAAMFLAEFVEDK.P
319	409	–	425	888.4231	1774.8316	1774.8084	13.1	0	120	1.1e-06	1	U	K.AGSSSAAMFLAEFVEDK.P + Oxidation (M)
347	409	–	427	1002.4821	2002.9496	2002.9346	7.46	0	110	1.3e-05	1	U	K.AGSSSAAMFLAEFVEDKPF.I
349	409	–	427	1010.4865	2018.9585	2018.9295	14.3	0	91	0.001	1	U	K.AGSSSAAMFLAEFVEDKPF.I + Oxidation (M)
397	418	–	439	1237.6858	2473.3570	2473.3257	12.7	0	82	0.0079	1	U	F.LAEFVEDKPFIHLDIAATAFVK.N
76	440	–	448	495.7639	989.5133	989.4964	17.0	0	34	3.2e+02	1	U	K.NTPTGVMVR.S + Oxidation (M)
92	449	–	457	518.3087	1034.6029	1034.6012	1.67	0	48	6.9	1	U	R.SLVEYILAK.Q

Band 7 45 kDa

2::Q4A9M4 **Mass:** 51376 **Score:** 3660 **Matches:** 98(42) **Sequences:** 51(23) **emPAI:** 25.54 tr|Q4A9M4|pepA Leucyl aminopeptidase Tax_Id=262719 [Mycoplasma hyopneumoniae (strain J / ATCC 25934 / NCTC 10110)]

Protein sequence coverage: 74%

1	**MKNFSEIFIK**	YSNKFESNR**I**	**TIEPAYSDSQ**	**IPMLIKEDLA**	**ITEYLGQK**KA
51	**YINLGSR**SKE	LTPNRFRKIA	AKLGHYPR**DM**	**QINFDKFPNS**	**FLRYLIEVIA**
101	**FQRSDIFSLR**	ADYAKNRAK**N**	**RDILVVSSYL**	**DELKPIIDKY**	**QIINNSVNYA**
151	**RYYQNMPPNM**	**ASSEFLASEI**	**QK**KMNLNPKL	TVKVLGENEV	R**KLGMNLLLA**
201	**VNR**GSTYDAK	**LVVISYEGLP**	**GSQYK**TAFIG	K**GITFDSGGY**	**NIKTGMYMND**
251	**MK**IDMSGAII	C**AAAIDALSQ**	**FNPLANVVAV**	**LPLTDNRLNG**	**DANTPDAVWR**
301	SMNGK**TVEIN**	**NTDAEGRLIL**	**ADAITYAIR**Q	EK**ASEIISIA**	**TLTGAIRIAL**
351	**GETFTGAFAN**	**EEK**IWKNFNE	ASK**EAGELIW**	**RMPLHQDFAQ**	**NIRD**SKVADL
401	KNTDFSGK**AG**	**SSSAAMFLAE**	**FVEDKPFIHL**	**DIAATAFVKN**	**TPTGVMVRSL**
451	**VEYILAK**QN				

Query	Start	–	End	Observed	Mr(expt)	Mr(calc)	ppm	M	Score	Expect	Rank	U	Peptide
102	1	–	10	636.8497	1271.6848	1271.6584	20.8	1	63	0.42	1	U	-.MKNFSEIFIK.Y + Oxidation (M)
50	3	–	10	499.2757	996.5369	996.5280	8.89	0	43	23	2	U	K.NFSEIFIK.Y
52	3	–	10	499.7687	997.5229	997.5120	10.9	0	62	0.33	1	U	K.NFSEIFIK.Y + Deamidated (NQ)
251	20	–	33	782.8866	1563.7587	1563.7490	6.19	0	56	2.2	1	U	R.ITIEPAYSDSQIPM.L
252	20	–	33	782.8893	1563.7640	1563.7490	9.57	0	53	4.7	1	U	R.ITIEPAYSDSQIPM.L
257	20	–	33	790.8884	1579.7623	1579.7440	11.6	0	66	0.24	1	U	R.ITIEPAYSDSQIPM.L + Oxidation (M)
314	20	–	36	960.0221	1918.0296	1918.0121	9.14	0	58	1.6	1	U	R.ITIEPAYSDSQIPMLIK.E
317	20	–	36	968.0205	1934.0264	1934.0070	10.0	0	58	1.8	1	U	R.ITIEPAYSDSQIPMLIK.E + Oxidation (M)
431	20	–	48	1099.2454	3294.7143	3294.6945	6.03	1	46	35	1	U	R.ITIEPAYSDSQIPMLIKEDLAITEYLGQK.K + Oxidation (M)
110	26	–	36	647.8488	1293.6831	1293.6638	14.8	0	71	0.058	1	U	A.YSDSQIPMLIK.E
118	26	–	36	655.8471	1309.6797	1309.6588	16.0	0	82	0.0045	1	U	A.YSDSQIPMLIK.E + Oxidation (M)
296	34	–	48	867.4992	1732.9838	1732.9611	13.1	1	66	0.22	1	U	M.LIKEDLAITEYLGQK.K
142	37	–	48	690.3745	1378.7345	1378.6980	26.5	0	47	17	1	U	K.EDLAITEYLGQK.K
143	37	–	48	690.4093	1378.8041	1378.6980	77.0	0	47	12	1	U	K.EDLAITEYLGQK.K
79	39	–	48	568.3344	1134.6543	1134.6284	22.7	0	55	1.9	1	U	D.LAITEYLGQK.K
27	50	–	57	447.2492	892.4839	892.4766	8.15	0	30	5.4e+02	3	U	K.AYINLGSR.S
28	50	–	57	447.2512	892.4879	892.4766	12.6	0	32	3.6e+02	3	U	K.AYINLGSR.S
55	79	–	86	505.7423	1009.4700	1009.4539	16.0	0	43	28	1	U	R.DMQINFDK.F
139	79	–	89	684.8210	1367.6275	1367.6180	6.98	1	59	0.84	1	U	R.DMQINFDKFPN.S
145	79	–	89	692.8195	1383.6244	1383.6129	8.30	1	51	6.1	1	U	R.DMQINFDKFPN.S + Oxidation (M)
269	79	–	91	801.8745	1601.7345	1601.7184	10.0	1	35	3e+02	1	U	R.DMQINFDKFPNSF.L
271	79	–	91	809.8728	1617.7311	1617.7133	10.9	1	60	1	1	U	R.DMQINFDKFPNSF.L + Oxidation (M)
305	79	–	93	936.9561	1871.8977	1871.8876	5.38	1	73	0.056	1	U	R.DMQINFDKFPNSFLR.Y + Deamidated (NQ)
308	79	–	93	944.4594	1886.9042	1886.8985	3.04	1	87	0.0025	1	U	R.DMQINFDKFPNSFLR.Y + Oxidation (M)
309	79	–	93	944.9628	1887.9111	1887.8825	15.1	1	75	0.034	1	U	R.DMQINFDKFPNSFLR.Y + Deamidated (NQ); Oxidation (M)
310	79	–	93	630.3130	1887.9171	1887.8825	18.3	1	47	22	1	U	R.DMQINFDKFPNSFLR.Y + Deamidated (NQ); Oxidation (M)
96	94	–	103	626.3640	1250.7135	1250.7023	8.96	0	76	0.013	1	U	R.YLIEVIAFQR.S
13	104	–	110	419.2330	836.4515	836.4392	14.7	0	55	1.4	1	U	R.SDIFSLR.A
373	120	–	139	777.4339	2329.2799	2329.2893	-4.01	1	64	0.43	1	U	K.NRDILVVSSYLDELKPIIDK.Y
374	120	–	139	1166.1411	2330.2677	2330.2733	-2.41	1	68	0.18	1	U	K.NRDILVVSSYLDELKPIIDK.Y + Deamidated (NQ)
337	122	–	139	1030.5807	2059.1469	2059.1452	0.78	0	74	0.043	1	U	R.DILVVSSYLDELKPIIDK.Y
338	122	–	139	1030.5837	2059.1529	2059.1452	3.73	0	42	67	2	U	R.DILVVSSYLDELKPIIDK.Y
339	122	–	139	1030.5898	2059.1651	2059.1452	9.64	0	50	8.8	1	U	R.DILVVSSYLDELKPIIDK.Y
359	122	–	140	1112.1305	2222.2465	2222.2086	17.0	1	67	0.22	1	U	R.DILVVSSYLDELKPIIDKY.Q
319	123	–	139	973.0758	1944.1371	1944.1183	9.69	0	64	0.31	1	U	D.ILVVSSYLDELKPIIDK.Y
175	128	–	139	717.3976	1432.7807	1432.7813	-0.42	0	93	0.00043	1	U	S.SYLDELKPIIDK.Y
130	129	–	139	673.8944	1345.7743	1345.7493	18.6	0	82	0.004	1	U	S.YLDELKPIIDK.Y
448	138	–	172	1382.3346	4143.9819	4143.9193	15.1	2	39	1.9e+02	1	U	I.DKYQIINNSVNYARYYQNMPPNMASSEFLASEIQK.K + 2 Deamidated (NQ); Oxidatio
183	140	–	151	727.8780	1453.7414	1453.7313	6.94	0	63	0.4	1	U	K.YQIINNSVNYAR.Y
184	140	–	151	727.8857	1453.7568	1453.7313	17.5	0	70	0.088	1	U	K.YQIINNSVNYAR.Y

Query	Start	–	End	Observed	Mr(expt)	Mr(calc)	ppm	M	Score	Expect	Rank	U	Peptide
187	140	–	151	728.3736	1454.7326	1454.7153	11.9	0	72	0.056	1	U	K.YQIINNSVNYAR.Y + Deamidated (NQ)
382	152	–	172	1224.5777	2447.1408	2447.1137	11.1	0	40	1.3e+02	1	U	R.YYQNMPPNMASSEFLASEIQK.K
386	152	–	172	1232.5784	2463.1423	2463.1086	13.7	0	56	3.6	1	U	R.YYQNMPPNMASSEFLASEIQK.K + Oxidation (M)
390	152	–	172	1240.5752	2479.1359	2479.1035	13.0	0	82	0.0089	1	U	R.YYQNMPPNMASSEFLASEIQK.K + 2 Oxidation (M)
306	156	–	172	940.4630	1878.9115	1878.8855	13.8	0	108	1.6e-05	1	U	N.MPPNMASSEFLASEIQK.K
311	156	–	172	948.4584	1894.9023	1894.8804	11.5	0	106	2.8e-05	1	U	N.MPPNMASSEFLASEIQK.K + Oxidation (M)
312	156	–	172	948.4643	1894.9140	1894.8804	17.7	0	59	1.3	1	U	N.MPPNMASSEFLASEIQK.K + Oxidation (M)
313	156	–	172	956.4584	1910.9022	1910.8754	14.0	0	80	0.01	1	U	N.MPPNMASSEFLASEIQK.K + 2 Oxidation (M)
177	160	–	172	720.8632	1439.7118	1439.6966	10.5	0	51	6.6	1	U	N.MASSEFLASEIQK.K
128	192	–	203	671.4071	1340.7996	1340.7962	2.55	1	90	0.00044	1	U	R.KLGMNLLLAVNR.G
129	192	–	203	671.9027	1341.7909	1341.7802	7.95	1	48	10	1	U	R.KLGMNLLLAVNR.G + Deamidated (NQ)
133	192	–	203	679.4060	1356.7974	1356.7911	4.64	1	88	0.00092	1	U	R.KLGMNLLLAVNR.G + Oxidation (M)
134	192	–	203	679.4158	1356.8171	1356.7911	19.2	1	88	0.0008	1	U	R.KLGMNLLLAVNR.G + Oxidation (M)
87	193	–	203	607.3629	1212.7113	1212.7012	8.27	0	92	0.00033	1	U	K.LGMNLLLAVNR.G
89	193	–	203	615.3640	1228.7135	1228.6962	14.1	0	91	0.00053	1	U	K.LGMNLLLAVNR.G + Oxidation (M)
90	193	–	203	615.3640	1228.7135	1228.6962	14.1	0	77	0.013	1	U	K.LGMNLLLAVNR.G + Oxidation (M)
273	211	–	225	826.9492	1651.8839	1651.8821	1.10	0	63	0.46	1	U	K.LVVISYEGLPGSQYK.T
274	211	–	225	826.9574	1651.9003	1651.8821	11.0	0	64	0.36	1	U	K.LVVISYEGLPGSQYK.T
100	232	–	243	636.3289	1270.6433	1270.6194	18.8	0	31	6e+02	1		K.GITFDSGGYNIK.T
101	232	–	243	636.3337	1270.6529	1270.6194	26.4	0	60	0.74	1		K.GITFDSGGYNIK.T
72	244	–	252	545.7318	1089.4490	1089.4293	18.0	0	23	2.2e+03	7	U	K.TGMYMNDMK.I
414	262	–	287	1347.2547	2692.4949	2692.4548	14.9	0	115	4.3e-06	1	U	C.AAAIDALSQFNPLANVVAVLPLTDNR.L
410	263	–	287	1311.7340	2621.4535	2621.4177	13.7	0	142	8.2e-09	1	U	A.AAIDALSQFNPLANVVAVLPLTDNR.L
404	264	–	287	1276.2076	2550.4006	2550.3806	7.85	0	96	0.00035	1	U	A.AIDALSQFNPLANVVAVLPLTDNR.L
366	267	–	287	1126.6505	2251.2864	2251.2325	24.0	0	118	1.5e-06	1	U	D.ALSQFNPLANVVAVLPLTDNR.L
264	273	–	287	796.4682	1590.9218	1590.9093	7.83	0	90	0.0007	1	U	N.PLANVVAVLPLTDNR.L
84	277	–	287	598.8649	1195.7153	1195.6925	19.1	0	64	0.14	1		N.VVAVLPLTDNR.L
168	288	–	300	714.8489	1427.6833	1427.6793	2.74	0	84	0.0032	1	U	R.LNGDANTPDAVWR.S
169	288	–	300	714.8590	1427.7035	1427.6793	16.9	0	90	0.00082	1	U	R.LNGDANTPDAVWR.S
170	288	–	300	715.3477	1428.6807	1428.6633	12.2	0	87	0.0019	1	U	R.LNGDANTPDAVWR.S + Deamidated (NQ)
171	288	–	300	715.3502	1428.6858	1428.6633	15.7	0	66	0.25	1	U	R.LNGDANTPDAVWR.S + Deamidated (NQ)
172	288	–	300	715.8567	1429.6989	1429.6474	36.0	0	58	1.4	1	U	R.LNGDANTPDAVWR.S + 2 Deamidated (NQ)
120	306	–	317	659.8295	1317.6444	1317.6161	21.5	0	76	0.023	1		K.TVEINNTDAEGR.L
124	318	–	329	666.9009	1331.7872	1331.7812	4.44	0	75	0.019	1		R.LILADAITYAIR.Q
125	318	–	329	666.9009	1331.7872	1331.7812	4.44	0	97	0.00013	1		R.LILADAITYAIR.Q
227	333	–	347	758.4447	1514.8749	1514.8668	5.39	0	111	5.8e-06	1	U	K.ASEIISIATLTGAIR.I
229	333	–	347	758.4500	1514.8854	1514.8668	12.3	0	123	3.5e-07	1	U	K.ASEIISIATLTGAIR.I
230	333	–	347	758.4526	1514.8906	1514.8668	15.7	0	70	0.061	1	U	K.ASEIISIATLTGAIR.I
231	333	–	347	758.4526	1514.8906	1514.8668	15.7	0	74	0.028	1	U	K.ASEIISIATLTGAIR.I
232	333	–	347	758.4526	1514.8906	1514.8668	15.7	0	90	0.00068	1	U	K.ASEIISIATLTGAIR.I
286	348	–	363	849.4246	1696.8347	1696.8308	2.28	0	95	0.00035	1	U	R.IALGETFTGAFANEEK.I
287	348	–	363	849.4274	1696.8402	1696.8308	5.54	0	100	9.6e-05	1	U	R.IALGETFTGAFANEEK.I
288	348	–	363	849.4329	1696.8512	1696.8308	12.0	0	78	0.015	1	U	R.IALGETFTGAFANEEK.I
289	348	–	363	849.4356	1696.8567	1696.8308	15.3	0	89	0.0014	1	U	R.IALGETFTGAFANEEK.I
40	374	–	381	487.2654	972.5162	972.5029	13.7	0	55	2.2	4		K.EAGELIWR.M
193	382	–	393	735.3760	1468.7375	1468.7245	8.84	0	67	0.17	1	U	R.MPLHQDFAQNIR.D
194	382	–	393	735.3786	1468.7426	1468.7245	12.3	0	27	2e+03	8	U	R.MPLHQDFAQNIR.D
209	382	–	393	743.3744	1484.7343	1484.7194	10.0	0	51	6.6	1	U	R.MPLHQDFAQNIR.D + Oxidation (M)
210	382	–	393	743.3770	1484.7395	1484.7194	13.5	0	74	0.037	1	U	R.MPLHQDFAQNIR.D + Oxidation (M)
211	382	–	393	743.3822	1484.7498	1484.7194	20.5	0	35	2.7e+02	3	U	R.MPLHQDFAQNIR.D + Oxidation (M)
213	382	–	393	743.8727	1485.7309	1485.7034	18.5	0	27	2e+03	1	U	R.MPLHQDFAQNIR.D + Deamidated (NQ); Oxidation (M)
260	382	–	394	792.8945	1583.7745	1583.7514	14.6	1	44	37	1	U	R.MPLHQDFAQNIRD.S

Query	Start	–	End	Observed	Mr(expt)	Mr(calc)	ppm	M	Score	Expect	Rank	U	Peptide
298	409	–	425	880.4213	1758.8281	1758.8134	8.35	0	102	6.8e-05	1	U	K.AGSSSAAMFLAEFVEDK.P
332	409	–	427	1002.5030	2002.9915	2002.9346	28.4	0	118	2e-06	1	U	K.AGSSSAAMFLAEFVEDKPF.I
334	409	–	427	1010.4805	2018.9464	2018.9295	8.38	0	96	0.00032	1	U	K.AGSSSAAMFLAEFVEDKPF.I + Oxidation (M)
389	418	–	439	825.4498	2473.3276	2473.3257	0.77	0	72	0.077	1	U	F.LAEFVEDKPFIHLDIAATAFVK.N
43	440	–	448	487.7671	973.5196	973.5015	18.6	0	27	1.7e+03	1	U	K.NTPTGVMVR.S
62	449	–	457	518.3130	1034.6115	1034.6012	10.0	0	48	6	1	U	R.SLVEYILAK.Q

Band 8 37kDa

2::Q4A9M4 **Mass:** 51376 **Score:** 1928 **Matches:** 46(17) **Sequences:** 27(10) **emPAI:** 2.90 tr|Q4A9M4|pepA Leucyl aminopeptidase Tax_Id=262719 [Mycoplasma hyopneumoniae (strain J / ATCC 25934 / NCTC 10110)]

Protein sequence coverage: 64%

**1**	MKNFSEIFIK	YSNKFESNRI	TIEPAYSDSQ	IPMLIKEDLA	ITEYLGQKKA
**51**	YINLGSRSKE	LTPNRFRKIA	AKLGHYPRDM	QINFDKFPNS	FLRYLIEVIA
**101**	FQRSDIFSLR	ADYAKNRAKN	RDILVVSSYL	DELKPIIDKY	QIINNSVNYA
**151**	RYYQNMPPNM	ASSEFLASEI	QKKMNLNPKL	TVKVLGENEV	RKLGMNLLLA
**201**	VNRGSTYDAK	LVVISYEGLP	GSQYKTAFIG	KGITFDSGGY	NIKTGMYMND
**251**	MKIDMSGAII	CAAAIDALSQ	FNPLANVVAV	LPLTDNRLNG	DANTPDAVWR
**301**	SMNGKTVEIN	NTDAEGRLIL	ADAITYAIRQ	EKASEIISIA	TLTGAIRIAL
**351**	GETFTGAFAN	EEKIWKNFNE	ASKEAGELIW	RMPLHQDFAQ	NIRDSKVADL
**401**	KNTDFSGKAG	SSSAAMFLAE	FVEDKPFIHL	DIAATAFVKN	TPTGVMVRSL
**451**	VEYILAKQN				

Query	Start	–	End	Observed	Mr(expt)	Mr(calc)	ppm	M	Score	Expect	Rank	U	Peptide
87	3	–	10	499.2799	996.5453	996.5280	17.4	0	42	34	3	U	K.NFSEIFIK.Y
238	20	–	33	782.8893	1563.7640	1563.7490	9.57	0	32	6.5e+02	10	U	R.ITIEPAYSDSQIPM.L
244	20	–	33	790.8857	1579.7569	1579.7440	8.22	0	45	27	1	U	R.ITIEPAYSDSQIPM.L + Oxidation (M)
280	20	–	36	960.0221	1918.0296	1918.0121	9.14	0	67	0.21	1	U	R.ITIEPAYSDSQIPMLIK.E
283	20	–	36	968.0234	1934.0323	1934.0070	13.1	0	59	1.4	1	U	R.ITIEPAYSDSQIPMLIK.E + Oxidation (M)
177	37	–	48	690.3621	1378.7096	1378.6980	8.43	0	66	0.22	1	U	K.EDLAITEYLGQK.K
67	50	–	57	447.2512	892.4879	892.4766	12.6	0	30	4.9e+02	4	U	K.AYINLGSR.S
178	79	–	89	692.8269	1383.6393	1383.6129	19.1	1	54	2.7	1	U	R.DMQINFDKFPN.S + Oxidation (M)
278	79	–	93	936.4693	1870.9241	1870.9036	11.0	1	107	2.2e-05	1	U	R.DMQINFDKFPNSFLR.Y
279	79	–	93	944.4681	1886.9217	1886.8985	12.3	1	96	0.00029	1	U	R.DMQINFDKFPNSFLR.Y + Oxidation (M)
63	87	–	93	440.7419	879.4693	879.4603	10.2	0	45	18	2	U	K.FPNSFLR.Y
153	94	–	103	626.3688	1250.7230	1250.7023	16.5	0	69	0.065	1	U	R.YLIEVIAFQR.S
40	104	–	110	419.2330	836.4515	836.4392	14.7	0	50	5	1	U	R.SDIFSLR.A
290	122	–	139	1030.5898	2059.1651	2059.1452	9.64	0	67	0.18	1	U	R.DILVVSSYLDELKPIIDK.Y
291	122	–	139	1030.5898	2059.1651	2059.1452	9.64	0	72	0.055	1	U	R.DILVVSSYLDELKPIIDK.Y
292	122	–	139	1030.5959	2059.1772	2059.1452	15.5	0	85	0.0029	1	U	R.DILVVSSYLDELKPIIDK.Y
196	140	–	151	727.8703	1453.7261	1453.7313	-3.60	0	55	2.5	1	U	K.YQIINNSVNYAR.Y
197	140	–	151	727.8805	1453.7465	1453.7313	10.5	0	72	0.057	1	U	K.YQIINNSVNYAR.Y
198	140	–	151	728.3685	1454.7224	1454.7153	4.87	0	49	12	5	U	K.YQIINNSVNYAR.Y + Deamidated (NQ)
199	140	–	151	728.3710	1454.7275	1454.7153	8.38	0	109	1.2e-05	1	U	K.YQIINNSVNYAR.Y + Deamidated (NQ)
200	140	–	151	728.3762	1454.7378	1454.7153	15.4	0	55	2.6	1	U	K.YQIINNSVNYAR.Y + Deamidated (NQ)

Query	Start	–	End	Observed	Mr(expt)	Mr(calc)	ppm	M	Score	Expect	Rank	U	Peptide
201	140	–	151	728.3813	1454.7480	1454.7153	22.4	0	61	0.75	1	U	K.YQIINNSVNYAR.Y + Deamidated (NQ)
316	152	–	172	1224.5810	2447.1474	2447.1137	13.8	0	54	5.7	1	U	R.YYQNMPPNMASSEFLASEIQK.K
319	152	–	172	1240.5785	2479.1425	2479.1035	15.7	0	61	1	1	U	R.YYQNMPPNMASSEFLASEIQK.K + 2 Oxidation (M)
171	192	–	203	671.4144	1340.8143	1340.7962	13.5	1	113	2.3e-06	1	U	R.KLGMNLLLAVNR.G
173	192	–	203	679.4109	1356.8073	1356.7911	11.9	1	94	0.00022	1	U	R.KLGMNLLLAVNR.G + Oxidation (M)
145	193	–	203	607.3676	1212.7206	1212.7012	16.0	0	94	0.00019	1	U	K.LGMNLLLAVNR.G
147	193	–	203	615.3664	1228.7182	1228.6962	18.0	0	103	3e-05	1	U	K.LGMNLLLAVNR.G + Oxidation (M)
255	211	–	225	826.9547	1651.8948	1651.8821	7.69	0	56	2.5	1	U	K.LVVISYEGLPGSQYK.T
156	232	–	243	636.3218	1270.6290	1270.6194	7.56	0	58	1.3	1		K.GITFDSGGYNIK.T
320	264	–	287	1276.2245	2550.4344	2550.3806	21.1	0	126	3.1e-07	1	U	A.AIDALSQFNPLANVVAVLPLTDNR.L
139	277	–	287	598.8626	1195.7106	1195.6925	15.2	0	64	0.14	1	U	N.VVAVLPLTDNR.L
190	288	–	300	714.8540	1427.6934	1427.6793	9.83	0	56	2.2	1	U	R.LNGDANTPDAVWR.S
191	288	–	300	714.8565	1427.6984	1427.6793	13.4	0	53	4.1	1	U	R.LNGDANTPDAVWR.S
192	288	–	300	715.3477	1428.6807	1428.6633	12.2	0	40	98	1	U	R.LNGDANTPDAVWR.S + Deamidated (NQ)
193	288	–	300	715.3477	1428.6807	1428.6633	12.2	0	99	0.00011	1	U	R.LNGDANTPDAVWR.S + Deamidated (NQ)
170	318	–	329	666.8886	1331.7627	1331.7812	-13.9	0	97	0.00016	1		R.LILADAITYAIR.Q
214	333	–	347	758.4500	1514.8854	1514.8668	12.3	0	74	0.026	1	U	K.ASEIISIATLTGAIR.I
215	333	–	347	758.4500	1514.8854	1514.8668	12.3	0	96	0.00019	1	U	K.ASEIISIATLTGAIR.I
216	333	–	347	758.4526	1514.8906	1514.8668	15.7	0	111	5.7e-06	1	U	K.ASEIISIATLTGAIR.I
217	333	–	347	758.4604	1514.9062	1514.8668	26.0	0	71	0.043	1	U	K.ASEIISIATLTGAIR.I
259	348	–	363	849.4356	1696.8567	1696.8308	15.3	0	99	0.00014	1	U	R.IALGETFTGAFANEEK.I
82	374	–	381	487.2654	972.5162	972.5029	13.7	0	56	1.9	3		K.EAGELIWR.M
208	382	–	393	743.3899	1484.7653	1484.7194	30.9	0	66	0.21	1	U	R.MPLHQDFAQNIR.D + Oxidation (M)
318	418	–	439	1237.6858	2473.3570	2473.3257	12.7	0	51	9.5	1	U	F.LAEFVEDKPFIHLDIAATAFVK.N
97	449	–	457	518.3152	1034.6158	1034.6012	14.2	0	36	1.1e+02	1	U	R.SLVEYILAK.Q

Band 9 27kDa

2::Q4A9M4 **Mass:** 51376 **Score:** 1560 **Matches:** 34(15) **Sequences:** 23(10) **emPAI:** 2.05 tr|Q4A9M4|pepA Leucyl aminopeptidase Tax_Id=262719 [Mycoplasma hyopneumoniae (strain J / ATCC 25934 / NCTC 10110)]

Protein sequence coverage: 55%

1	MK**NFSEIFIK**	YSNKFESNR**I**	**TIEPAYSDSQ**	**IPMLIKEDLA**	**ITEYLGQK**KA
51	**YINLGSR**SKE	LTPNRFRKIA	AKLGHYPR**DM**	**QINFDKFPNS**	**FLRYLIEVIA**
101	**FQRSDIFSLR**	ADYAKNRAKN	R**DILVVSSYL**	**DELKPIIDKY**	**QIINNSVNYA**
151	**RYYQNMPPNM**	**ASSEFLASEI**	**QK**KMNLNPKL	TVKVLGENEV	R**KLGMNLLLA**
201	**VNR**GSTYDAK	**LVVISYEGLP**	**GSQYK**TAFIG	K**GITFDSGGY**	**NIK**TGMYMND
251	MKIDMSGAII	CAAAIDALSQ	FNPLAN**VVAV**	**LPLTDNRLNG**	**DANTPDAVWR**
301	SMNGKTVEIN	NTDAEGR**LIL**	**ADAITYAIR**Q	EK**ASEIISIA**	**TLTGAIRIAL**
351	**GETFTGAFAN**	**EEK**IWKNFNE	ASKEAGELIW	R**MPLHQDFAQ**	**NIR**DSKVADL
401	KNTDFSGKAG	SSSAAMFLAE	FVEDKPFIHL	DIAATAFVKN	TPTGVMVR**SL**
451	**VEYILAK**QN				

Query	Start	–	End	Observed	Mr(expt)	Mr(calc)	ppm	M	Score	Expect	Rank	U	Peptide
77	3	–	10	499.2778	996.5411	996.5280	13.1	0	39	67	1	U	K.NFSEIFIK.Y
197	20	–	33	782.8893	1563.7640	1563.7490	9.57	0	42	60	1	U	R.ITIEPAYSDSQIPM.L
202	20	–	33	790.8831	1579.7516	1579.7440	4.85	0	30	8.4e+02	7	U	R.ITIEPAYSDSQIPM.L + Oxidation (M)

Query	Start	–	End	Observed	Mr(expt)	Mr(calc)	ppm	M	Score	Expect	Rank	U	Peptide
232	20	–	36	960.0250	1918.0355	1918.0121	12.2	0	47	23	1	U	R.ITIEPAYSDSQIPMLIK.E
234	20	–	36	968.0293	1934.0441	1934.0070	19.2	0	53	6	1	U	R.ITIEPAYSDSQIPMLIK.E + Oxidation (M)
147	37	–	48	690.3621	1378.7096	1378.6980	8.43	0	50	9.5	2	U	K.EDLAITEYLGQK.K
52	50	–	57	447.2532	892.4919	892.4766	17.1	0	39	62	1	U	K.AYINLGSR.S
145	79	–	89	684.8235	1367.6325	1367.6180	10.6	1	62	0.45	1	U	R.DMQINFDKFPN.S
230	79	–	93	944.4681	1886.9217	1886.8985	12.3	1	94	0.00046	1	U	R.DMQINFDKFPNSFLR.Y + Oxidation (M)
120	94	–	103	626.3688	1250.7230	1250.7023	16.5	0	89	0.00069	1	U	R.YLIEVIAFQR.S
26	104	–	110	419.2350	836.4554	836.4392	19.3	0	51	3.6	1	U	R.SDIFSLR.A
242	122	–	139	1030.5837	2059.1529	2059.1452	3.73	0	74	0.041	1	U	R.DILVVSSYLDELKPIIDK.Y
243	122	–	139	1030.5959	2059.1772	2059.1452	15.5	0	85	0.0027	1	U	R.DILVVSSYLDELKPIIDK.Y
244	122	–	139	1030.5989	2059.1833	2059.1452	18.5	0	74	0.036	1	U	R.DILVVSSYLDELKPIIDK.Y
245	122	–	139	1030.6020	2059.1894	2059.1452	21.4	0	61	0.63	1	U	R.DILVVSSYLDELKPIIDK.Y
164	140	–	151	727.8780	1453.7414	1453.7313	6.94	0	74	0.038	1	U	K.YQIINNSVNYAR.Y
165	140	–	151	727.8857	1453.7568	1453.7313	17.5	0	86	0.0021	1	U	K.YQIINNSVNYAR.Y
166	140	–	151	728.3685	1454.7224	1454.7153	4.87	0	55	2.7	1	U	K.YQIINNSVNYAR.Y + Deamidated (NQ)
167	140	–	151	728.3736	1454.7326	1454.7153	11.9	0	54	3.3	1	U	K.YQIINNSVNYAR.Y + Deamidated (NQ)
168	140	–	151	728.3787	1454.7429	1454.7153	18.9	0	73	0.044	1	U	K.YQIINNSVNYAR.Y + Deamidated (NQ)
268	152	–	172	1224.5777	2447.1408	2447.1137	11.1	0	59	1.6	1	U	R.YYQNMPPNMASSEFLASEIQK.K
269	152	–	172	1240.5852	2479.1559	2479.1035	21.1	0	72	0.087	1	U	R.YYQNMPPNMASSEFLASEIQK.K + 2 Oxidation (M)
140	192	–	203	671.4169	1340.8192	1340.7962	17.2	1	113	2.2e-06	1	U	R.KLGMNLLLAVNR.G
143	192	–	203	679.4134	1356.8122	1356.7911	15.5	1	95	0.00019	1	U	R.KLGMNLLLAVNR.G + Oxidation (M)
118	193	–	203	615.3664	1228.7182	1228.6962	18.0	0	66	0.15	1	U	K.LGMNLLLAVNR.G + Oxidation (M)
207	211	–	225	826.9601	1651.9057	1651.8821	14.3	0	47	18	1	U	K.LVVISYEGLPGSQYK.T
124	232	–	243	636.3242	1270.6337	1270.6194	11.3	0	49	11	1		K.GITFDSGGYNIK.T
114	277	–	287	598.8626	1195.7106	1195.6925	15.2	0	74	0.015	1	U	N.VVAVLPLTDNR.L
160	288	–	300	715.3527	1428.6909	1428.6633	19.3	0	77	0.02	1	U	R.LNGDANTPDAVWR.S + Deamidated (NQ)
139	318	–	329	666.9082	1331.8018	1331.7812	15.5	0	89	0.00057	1		R.LILADAITYAIR.Q
182	333	–	347	758.4500	1514.8854	1514.8668	12.3	0	126	1.9e-07	1	U	K.ASEIISIATLTGAIR.I
210	348	–	363	849.4329	1696.8512	1696.8308	12.0	0	92	0.00063	1	U	R.IALGETFTGAFANEEK.I
170	382	–	393	735.3812	1468.7478	1468.7245	15.8	0	51	6.8	2	U	R.MPLHQDFAQNIR.D
88	449	–	457	518.3195	1034.6245	1034.6012	22.5	0	45	12	1	U	R.SLVEYILAK.Q

Band 10 25kDa

2::Q4A9M4 **Mass:** 51376 **Score:** 587 **Matches:** 9(3) **Sequences:** 9(3) tr|Q4A9M4|pepA Leucyl aminopeptidase Tax_Id=262719 [Mycoplasma hyopneumoniae (strain J / ATCC 25934 / NCTC 10110)]

Protein sequence coverage: 27%

**1**	MKNFSEIFIK	YSNKFESNR**I**	**TIEPAYSDSQ**	**IPMLIKEDLA**	**ITEYLGQK**KA
**51**	YINLGSRSKE	LTPNRFRKIA	AKLGHYPRDM	QINFDKFPNS	FLR**YLIEVIA**
**101**	**FQR**SDIFSLR	ADYAKNRAKN	R**DILVVSSYL**	**DELKPIIDK**Y	QIINNSVNYA
**151**	RYYQNMPPNM	ASSEFLASEI	QKKMNLNPKL	TVKVLGENEV	RK**LGMNLLLA**
**201**	**VNR**GSTYDAK	**LVVISYEGLP**	**GSQYK**TAFIG	KGITFDSGGY	NIKTGMYMND
**251**	MKIDMSGAII	CAAAIDALSQ	FNPLANVVAV	LPLTDNRLNG	DANTPDAVWR
**301**	SMNGKTVEIN	NTDAEGR**LIL**	**ADAITYAIR**Q	EK**ASEIISIA**	**TLTGAIRIAL**
**351**	**GETFTGAFAN**	**EEK**IWKNFNE	ASKEAGELIW	RMPLHQDFAQ	NIRDSKVADL
**401**	KNTDFSGKAG	SSSAAMFLAE	FVEDKPFIHL	DIAATAFVKN	TPTGVMVRSL

**451**	VEYILAKQN

Query	Start	–	End	Observed	Mr(expt)	Mr(calc)	ppm	M	Score	Expect	Rank	U	Peptide
37	20	–	36	968.0293	1934.0441	1934.0070	19.2	0	68	0.16	1	U	R.ITIEPAYSDSQIPMLIK.E + Oxidation (M)
26	37	–	48	690.3745	1378.7345	1378.6980	26.5	0	54	3.6	2	U	K.EDLAITEYLGQK.K
24	94	–	103	626.3711	1250.7277	1250.7023	20.3	0	64	0.23	2	U	R.YLIEVIAFQR.S
40	122	–	139	687.4001	2059.1785	2059.1452	16.1	0	56	2.1	1	U	R.DILVVSSYLDELKPIIDK.Y
22	193	–	203	607.3652	1212.7159	1212.7012	12.1	0	71	0.041	1	U	K.LGMNLLLAVNR.G
35	211	–	225	826.9629	1651.9111	1651.8821	17.6	0	25	2.8e+03	2	U	K.LVVISYEGLPGSQYK.T
25	318	–	329	666.9131	1331.8116	1331.7812	22.8	0	63	0.21	1	U	R.LILADAITYAIR.Q
28	333	–	347	758.4526	1514.8906	1514.8668	15.7	0	112	4e-06	1	U	K.ASEIISIATLTGAIR.I
36	348	–	363	849.4412	1696.8678	1696.8308	21.8	0	88	0.0015	1	U	R.IALGETFTGAFANEEK.I

Band 11 20kDa

Protein sequence coverage: 17%

**1**	MKNFSEIFIK	YSNKFESNRI	TIEPAYSDSQ	IPMLIKEDLA	ITEYLGQKKA
**51**	YINLGSRSKE	LTPNRFRKIA	AKLGHYPRDM	QINFDKFPNS	FLR**YLIEVIA**
**101**	**FQR**SDIFSLR	ADYAKNRAKN	R**DILVVSSYL**	**DELKPIIDK**Y	QIINNSVNYA
**151**	RYYQNMPPNM	ASSEFLASEI	QKKMNLNPKL	TVKVLGENEV	RKLGMNLLLA
**201**	VNRGSTYDAK	LVVISYEGLP	GSQYKTAFIG	KGITFDSGGY	NIKTGMYMND
**251**	MKIDMSGAII	CAAAIDALSQ	FNPLANVVAV	LPLTDNRLNG	DANTPDAVWR
**301**	SMNGKTVEIN	NTDAEGR**LIL**	**ADAITYAIR**Q	EK**ASEIISIA**	**TLTGAIRIAL**
**351**	**GETFTGAFAN**	**EEK**IWKNFNE	ASKEAGELIW	RMPLHQDFAQ	NIRDSKVADL
**401**	KNTDFSGKAG	SSSAAMFLAE	FVEDKPFIHL	DIAATAFVKN	TPTGVMVR**SL**
**451**	**VEYILAK**QN				

Query	Start	–	End	Observed	Mr(expt)	Mr(calc)	ppm	M	Score	Expect	Rank	U	Peptide
7	94	–	103	626.3759	1250.7372	1250.7023	27.9	0	62	0.32	1	U	R.YLIEVIAFQR.S
14	122	–	139	1030.6111	2059.2076	2059.1452	30.3	0	39	96	8	U	R.DILVVSSYLDELKPIIDK.Y
8	318	–	329	666.9131	1331.8116	1331.7812	22.8	0	56	1.1	1	U	R.LILADAITYAIR.Q
10	333	–	347	758.4578	1514.9010	1514.8668	22.6	0	99	8.1e-05	1	U	K.ASEIISIATLTGAIR.I
12	348	–	363	849.4412	1696.8678	1696.8308	21.8	0	64	0.39	1	U	R.IALGETFTGAFANEEK.I
5	449	–	457	518.3195	1034.6245	1034.6012	22.5	0	27	7.5e+02	1	U	R.SLVEYILAK.Q
