# Supplementary figures and images for: MHJ_0461 is a multifunctional leucine aminopeptidase on the surface of Mycoplasma hyopneumoniae
Source: Open Biol. 2015 Jan 14;5(1):140175. doi: 10.1098/rsob.140175 (PMC4313372; doi:10.1098/rsob.140175)

Ligand blot of rMHJ 0461 probed with plasminogen in the presence of a lysine analog shows no binding

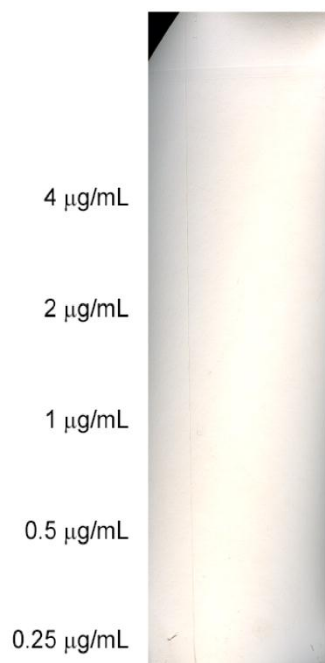

Supplement: rMHJ_0461 does not bind plasminogen in the presence of a lysine analogue [file rsob140175supp3.pdf]
